# Equilibrium Solubility and Solution Structure of Paracetamol: A Molecular Dynamics Study Spanning Four Orders of Magnitude in Diverse Pure Solvents

**DOI:** 10.1002/cphc.70593

**Published:** 2026-10-22

**Authors:** Katherine E. Mevis, Ella M. Smith, Andrew S. Paluch

**Affiliations:** ^1^ Department of Chemical, Paper, and Biomedical Engineering Miami University Oxford Ohio USA

**Keywords:** free energy, molecular dynamics, solubility, solvation structure, thermodynamics

## Abstract

Molecular dynamics simulations are used to investigate the equilibrium solubility and solution structure of paracetamol (or acetaminophen) in water, ethanol, acetone, diethylamine, dimethylformamide, dimethyl sulfoxide, toluene, and cyclohexane. A rigorous thermodynamic framework relates the solution‐phase concentration‐dependent number density and solvation free energy to the pure crystalline excess Gibbs free energy, predicting solubility across four orders of magnitude. Results for ethanol agree with experimental data and reference simulations. The infinite dilution approximation is established as a rapid diagnostic tool and strategic method for staging simulations, especially for sparingly soluble systems where standard simulations are inherently supersaturated. In diethylamine, the model under‐predicted solubility by two orders of magnitude. Structural analysis reveals that underestimating solvent‐solute hydrogen bonding leads to unphysical aggregation, illustrating the critical distinction between experimental and model equilibrium. This work highlights phase equilibria expressions as essential diagnostics for the future parameterization of force fields for complex, multifunctional molecules.

## Graphical abstract

We present rigorous solubility predictions for the molecular solute paracetamol across diverse solvents spanning four orders of magnitude. Complemented by detailed structural analysis, we link microscopic evolution to macroscopic solubility plots. This work also investigates the utility of the infinite dilution approximation as a diagnostic tool for generating initial guesses and staging computationally demanding simulations.

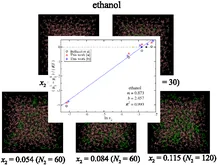

## 1 Introduction

The annual research and development expenditures aimed at discovering new drugs and medications by U.S. pharmaceutical companies have continued to increase, with a total of $83 billion in 2019 [1]. Typically, moving a drug from concept to market can take between 10 and 15 years and cost as much as $1 billion [2]. Despite this large investment, for the decade 2000–2009, only a median of 25 drugs were approved by the U.S. Food and Drug Administration (FDA) each year. Over the following decade, 2010–2019, this increased modestly to 40 drugs per year [3]. In 2007, approximately 2.42 million people died in the U.S. from disease [4]. In 2019, this increased to 2.85 million [5]. Advancements in drug design methods, methods to produce drugs at an industrial scale, and methods to formulate the drug for delivery are critical to improving the rate of survival from disease. The FDA's Critical Path Initiative (CPI), issued in 2004, emphasizes the inefficiencies of conventional design schemes and advocates the need for novel approaches, including the use of computational methods [6].

During the drug development process, following discovery, clinical trials (to determine efficacy, safety, and dosage) and research and development related to the manufacture of the drug are commonly performed in parallel to shorten the time to market. A drug needs to satisfy various solubility criteria before it may be manufactured at an industrial scale at a reasonable expense and then formulated for delivery [7, 8, 9]. Solvent selection is critical. With regard to production, drug synthesis is carried out in solution. Once synthesized, the drug must be separated and purified. Alternatively, for natural compounds, it may be necessary to selectively extract the desired compound from its natural environment. Subsequently, the drug needs to be formulated for delivery. For example, if a tablet is to be made to be administered orally, solvents will be necessary for the crystallization process. Each of these processes can have different solubility requirements, complicating the design. In a typical manufacturing process, solvents account for 80%–90% of the utilized material [7, 9]. As a consequence of the large and complex chemistries of drug molecules, finding suitable solvents for each stage of the manufacturing process is challenging. Early in the development process, this is further complicated by the limited availability of experimental solubility data.

Given the importance of being able to predict the solubility of pharmaceuticals, considerable work has been done in this area. We can place this work into three camps. First are efficient analytic‐based equations. Promising predictions have been made using the theoretically based NRTL‐SAC model [10], MOSCED model [11], and the perturbed‐chain statistical associating fluid theory (PC‐SAFT) equation of state [12, 13, 14, 15]. However, these models routinely rely on experimental reference data to determine the necessary model parameters for the solute or mixing rule. For the case of the relatively highly parameterized PC‐SAFT, the model routinely relies on experimental reference data, such as neat liquid densities and vapor pressures, to determine necessary pure‐substance parameters [14]. Furthermore, for complex mixtures, the application of an empirical binary interaction parameter ($k_{ij}$) is often required to achieve reasonable results, complicating its use for the design of novel pharmaceuticals and unconventional solvent environments where such data is unavailable [14].

A related and increasingly popular alternative is the Conductor‐like Screening Model for Real Solvents (COSMO‐RS), which serves as a unique bridge between quantum mechanics (QM) and statistical thermodynamics [14, 16]. Rather than relying on classical group contributions or fitted parameters, COSMO‐RS utilizes QM calculations to generate a substance‐specific molecular surface charge density distribution, known as a *σ*‐profile. This profile is then used to model intermolecular interactions, including hydrogen bonding, via a statistical thermodynamic procedure [14, 16]. While COSMO‐RS is often described as “first‐principles‐aided” and can be applied a priori to novel molecules, it nonetheless leverages a fixed set of substance‐independent universal parameters [14]. Recent benchmarks by Klajmon [14] demonstrate that while COSMO‐RS often provides superior qualitative solvent ranking compared to “pure” (parameter‐free) PC‐SAFT, both frameworks continue to face significant challenges in achieving quantitative accuracy, often struggling to correctly predict the magnitude and direction of deviations from ideality for complex, multifunctional drug molecules.

Second is the use of molecular simulation [17, 18, 19, 20, 21, 22, 23, 24, 25, 26]. With molecular simulation, one may not only predict solubility devoid of reference data, but molecular simulation may additionally be used to probe the underlying intermolecular interactions. Most molecular simulation studies employ a “direct” approach, wherein they leverage the rigorous phase equilibrium requirement that the chemical potential of the pure crystalline solid solute is equal to the chemical potential of the solute in the solution. This typically involves two stages [17, 18, 19, 20, 24]: (1) determination of the chemical potential (or Gibbs free energy) of the pure crystalline solute and (2) determining the concentration‐dependent chemical potential of the solute in solution. If we are interested in predicting solubility in a range of solvents, the chemical potential of the pure crystalline solute is a pure component property and needs only be computed once. On the other hand, the concentration‐dependent chemical potential of the solute in solution must be computed in each solvent. While rigorously correct, a shortcoming of molecular simulation for design applications is its limited computational efficiency of the solution‐phase calculations, in addition to the accuracy of the intermolecular potential used.

Commonly, if one has an estimate of the equilibrium solubility, the necessary solution‐phase solvation free energy calculations may be staged and performed in parallel to obtain the concentration‐dependent chemical potential [17, 18, 19]. With adequate computational resources, predictions may be efficiently made in real time.

For the case of sparingly soluble solutes, the number of solution‐phase solvation free‐energy calculations can be reduced to one through the application of the “infinite dilution” approximation [20, 21, 22, 23]. For this case, we perform a solvation free‐energy calculation only at the infinite dilution limit (i.e., a single solute molecule in solution). We then extrapolate the solution‐phase chemical potential by assuming that the solvation free energy (or equivalently the residual or excess contribution to the chemical potential) is constant and equal to the value at infinite dilution. This approach is analogous to the application of Henry's law in the modeling of the solubility of noncondensable gases. The calculations at the infinite dilution limit capture solute–solvent and solvent–solvent interactions. We can therefore view the infinite dilution approximation as assuming that solute–solute interactions are negligible, which would otherwise be captured in composition‐dependent calculations. Closely related, a “dilute solution” approximation has been proposed [21, 22, 23]. For this case, solute–solute interactions are assumed negligible, and a free‐energy calculation is performed only at the infinite dilution limit. The difference is that rather than assumng that the solvation free energy is constant, the difference relative to the infinite dilution limit is modeled using an athermal (or combinatorial) excess Gibbs free energy model, such as the Flory–Huggins equation.

Recently, Cheng [27] presented an efficient, thermodynamically sound method to compute the solution‐phase composition‐dependent chemical potential requiring a solvation free energy calculation only in the infinite dilution limit. Solvation free‐energy calculations at finite concentrations are replaced with efficient equilibrium simulations of the system. Static structure factors are used to compute the chemical potential at a finite concentration relative to the infinite dilution limit, where the value at the infinite dilution limit is obtained using the single‐solvation free energy calculation. This approach was subsequently leveraged by Reinhardt et al. [24] in the context of solubility predictions.

An alternative method that has been used in the direct approach is osmotic ensemble Monte Carlo (OEMC) [25]. For this case, the chemical potential of the pure crystalline solute is first determined. OEMC is an open ensemble used to model the solution phase, where the chemical potential of the solute can be imposed, and the solute concentration is allowed to fluctuate until equilibrium is established.

Finally, Schaefer and Kohns [26] recently demonstrated a strategy for electrolyte systems that avoids the use of solution‐phase free energy calculations altogether and additionally does not require one to model the pure crystalline solute. In that work, they demonstrated that the formation of stable ion clusters (e.g., clusters of six or more ions in salt solutions) can serve as a rapid diagnostic for the onset of phase transitions, providing a simple post‐processing tool to identify the transition from stable to supersaturated states. The approach requires only a series of solution‐phase equilibrium simulations covering a range of concentrations of interest.

A critical challenge in molecular modeling is the selection of experimental benchmarks used to parameterize the underlying intermolecular potentials. Traditionally, many classical force fields have been optimized using pure component properties, such as neat liquid densities and heats of vaporization [28]. However, this approach often fails to capture the subtle interspecies associations found in mixtures, particularly for molecules like acetone that function as strong hydrogen‐bond acceptors but not donors. When parameterized solely against pure‐liquid data, these models may lack the necessary features to describe unlike interactions. For example, Kamath et al. [29] demonstrated that standard OPLS and TraPPE models failed to reproduce the experimental minimum pressure azeotrope in acetone‐chloroform mixtures because they underestimated the intermolecular association between the carbonyl oxygen and the chloroform hydrogen. Similarly, Weerasinghe and Smith [30] observed that standard models poorly reproduced the self‐aggregation and thermodynamic activity in acetone‐water mixtures, leading to the development of a Kirkwood–Buff‐derived force field trained specifically against mixture‐phase activity data. These findings underscore the limitations of pure‐component benchmarks and have informed modern efforts like the OpenFF Sage 2.0.0 force field, which, for the first time, refit Lennard–Jones (LJ) parameters against liquid densities and enthalpies of mixing to better describe interactions between unlike chemistries [31]. Analogously, Jorgensen and coworkers [32, 33] leveraged mixture properties by using experimental free energies of hydration to determine optimal scaling factors for quantum‐mechanical charge models, such as CM5, to account for condensed‐phase polarization. Collectively, these studies suggest that incorporating phase equilibria and mixture data, analogous to the use of vapor–liquid equilibrium (VLE) in the parameterization of the TraPPE force field [34, 35], is essential for developing robust molecular models capable of describing the complex solvation environments of drug‐like molecules.

More recently, an emerging third camp for solubility prediction has utilized machine learning (ML) and data‐driven quantitative structure–property relationship (QSPR) models to predict solubility [36, 37, 38]. While these approaches were traditionally hampered by a reliance on aqueous‐only training sets, the curation of expansive, multi‐solvent databases like AqSolDB and BigSolDB has enabled new workflows capable of identifying optimal organic cosolvents for hydrophobic molecules [38]. Furthermore, the field is actively addressing the “black box” nature of early ML efforts by adopting interpretable graph representation learning and molecule attention transformers [37, 37]. These architectures allow researchers to use techniques such as layer‐wise relevance propagation (LRP) to visualize how specific molecular motifs, such as high‐polarity regions, drive a prediction, aligning the results with chemical intuition. Notably, recent hybrid approaches have begun to bridge the gap between data‐driven and physics‐based models [37]. For instance, Ge and Ji [36] successfully utilized ML to estimate the pure‐component parameters required for the PC‐SAFT equation of state, attempting to combine the predictive speed of ML with the thermodynamic consistency of a rigorous equation of state. Despite these strides, achieving robust quantitative accuracy for novel, unseen solutes remains a significant hurdle as models often see performance drops when applied outside their training distributions [36, 38]. This maintains the necessity of first‐principles methods to provide the reliable physical insights required for early‐stage process design.

However, molecular simulation and ML are increasingly viewed as complementary tools. For instance, first‐principles simulations can generate high‐quality surrogate data to augment training sets in chemical regions where experimental data is sparse or serve as a rigorous validation layer for top‐performing candidates identified during rapid ML‐based virtual screenings [36, 39, 40]. Furthermore, detailed structural analyses, such as the investigation of the solvent structure at saturation performed in the present work, can identify critical intermolecular motifs to be used as physically grounded descriptors in future data‐driven models, bridging the gap between predictive speed and mechanistic interpretability [36].

In the present work, we build upon the rigorous thermodynamic framework established by Belluci et al. [18] to investigate the equilibrium solubility and solution structure of paracetamol across eight pure solvents representing a broad range of chemical functionalities. By considering solvents that span four orders of magnitude in experimental solubility, from the highly soluble dimethyl sulfoxide (DMSO) to sparingly soluble cyclohexane, we assess the universal applicability of the fixed‐charge force field and the underlying thermodynamic models. Specifically, we evaluate the utility of the infinite dilution approximation as an efficient diagnostic tool and a strategic method for staging simulations, particularly for systems where standard equilibrium simulations are inherently supersaturated. We further employ a comprehensive structural analysis using connection matrices and radial distribution functions (RDFs) to diagnose the molecular basis for discrepancies between simulation and experiment. This investigation focuses particularly on complex environments, such as diethylamine (DEAM), to distinguish between experimental and model equilibrium and to provide a clear route for the future parameterization of force fields for multifunctional drug‐like molecules.

## 2 Methodology

### 2.1 Computational Details

#### 2.1.1 Molecular Simulation

All interactions were modeled using a “class I” potential energy function where all nonbonded intermolecular interactions $\left(U_{\mathrm{nb}}\right)$ were accounted for using a combined LJ plus fixed point charge model of the form [41, 42]

(1) $$U_{\mathrm{nb}}\left(r_{ij}\right)=4\varepsilon_{ij}\left[{\left(\frac{\sigma_{ij}}{r_{ij}}\right)}^{12}-{\left(\frac{\sigma_{ij}}{r_{ij}}\right)}^{6}\right]+\frac{1}{4\pi \varepsilon_{0}}\frac{q_{i}q_{j}}{r_{ij}}$$

where $r_{ij}$ is the separation distance between sites i and j, $\varepsilon_{ij}$ is the well‐depth of the LJ potential, $\sigma_{ij}$ is the distance at which the LJ potential is zero, and $q_{i}$ and $q_{j}$ are the partial charges of sites i and j, respectively. Simulations were performed for paracetamol in water, ethanol, acetone, diethylamine (DEAM), dimethylformamide (DMF), dimethyl sulfoxide (DMSO), toluene, and cyclohexane (see Figure 1). The solvents were selected due to the availability of experimental solubility data, with paracetamol exhibiting a wide range of mole fraction solubilities spanning four orders of magnitude ($10^{-5}-10^{-1}$) in addition to their range of chemical functionalities [43, 44]. The work of Belluci et al. [18] used molecular simulations to predict the equilibrium solubility of paracetamol in ethanol and serves as the basis of this work. The inclusion of ethanol allowed us to first ensure our results were consistent with the work of Belluci et al. [18].

**FIGURE 1:**
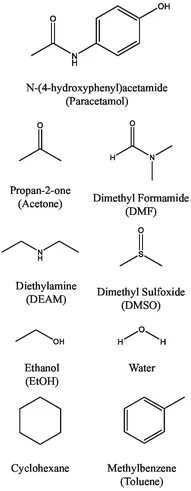
Chemical structure of the studied solute (paracetamol or acetaminophen, CAS: 103‐90‐2) and solvents as labeled.

Force fields were selected following the work of Belluci et al. [18]. In that work, CGenFF (CHARMM General Force Field) with CHARMM36 parameters was used to model ethanol [45, 46, 47, 48, 49]; paracetamol was modeled using a re‐optimized CGenFF with CHARMM36 parameters from their earlier work, where the partial charges were changed to minimize deviations from reference values of the lattice energy of polymorph form I relative to form II, the heat of sublimation, heat of vaporization, heat of fusion, and the solvation free energy in pure ethanol [18, 50]. Consistent with Belluci et al. [18] we generated force field files using CGenFF for paracetamol and the studied organic solvents. To accomplish this, first single‐molecule 3D structures were generated with Open Babel 2.3.2 [51, 52] by performing a systematic conformation search to identify the lowest‐energy conformer, followed by geometry optimization. For each molecule, the 3D structure (mol2 file) was then uploaded to the online CGenFF program version 2.5 (for use with CGenFF version 4.6), which produced a CHARMM toppar stream file (str file) [53]. After confirming there were no issues, the available Python script was used to convert the CGenFF toppar stream file from CHARMM to GROMACS [54, 55, 56, 57] format (cgenff_charmm2gmx.py) with the CHARMM36 force field files for GROMACS (charmm36‐jul2021.ff) [58]. For paracetamol, the partial charges were subsequently updated to match those used by Belluci et al. [18, 50]. Water was modeled using the rigid TIP4P/2005 model [59].

Molecular dynamics were used to sample configurational phase space. For all of the systems, the number of molecules was chosen to obtain a cubic box with an edge length of approximately 4.8 nm at 298 K and 1 bar. For water and the organic solvents, we considered the case of a single paracetamol molecule infinitely dilute in solution. For ethanol, DMSO, DMF, diethylamine, and acetone, simulations were performed at three or four additional concentrations of paracetamol in equal increments from infinite dilution to beyond the experimental solubility. An additional set of simulations for ethanol was performed using the same system compositions as those in Belluci et al. [18] for comparison.

Initial structures were generated using Packmol [60, 61]. This was followed by 3000 steepest descent minimization steps to remove any bad contacts that might have resulted from the packing. We next performed two steps of equilibration in an NPT ensemble at 298 K and 1 bar with the equations of motion integrated using the Verlet leap‐frog algorithm [41, 42, 62, 63]. First, we performed a 2 ns equilibration using the Berendsen thermostat and a barostat [62, 63, 64]. This was followed by 12 ns of equilibration using the stochastic velocity rescaling thermostat [62, 65, 66, 67] and the Parrinello–Rahman barostat [68]. The final structure from this series of simulations was then used as the initial structure for our free‐energy calculations, as will be described momentarily. The final NPT equilibration for paracetamol in ethanol, DMSO, DMF, diethylamine, and acetone (at each concentration) was continued for an additional 100 ns, which was used for subsequent structural analysis.

All of the molecular dynamics simulations were performed using GROMACS 2020.2 [54, 55, 56, 57]. For paracetamol and the organic solvents, all bond lengths involving hydrogen were constrained using P‐LINCS [62, 69, 70]. Water was modeled as completely rigid using the SETTLE algorithm [71, 72]. The Verlet neighbor list was used, and LJ interactions were cut off at 1.5 nm with long‐range analytic dispersion corrections applied to the energy and pressure [41, 42, 62, 63]. Lorentz–Berthelot mixing rules were used for unlike LJ sites [41]. The electrostatic terms were evaluated using the smooth particle‐mesh Ewald (SPME) method with tin‐foil boundary conditions [62, 63, 73] and real‐space interactions truncated at 1.5 nm. The SPME B‐spline was order 4, the Fourier spacing was 0.12 nm, and the relative tolerance between long‐ and short‐range energies was 10^−8^. The equations of motion were integrated with a timestep of 2 fs, the time constant for the thermostat was 1 ps, and the time constant for the barostat was 4 ps.

All of the GROMACS force field files used in the present study, along with sample GROMACS input files, are provided in the Supporting Information accompanying the electronic version of this article.

### 2.2 Free Energy Calculations

The solvation free energy, $\Delta G_{2}^{\mathrm{solv}}$, for paracetamol in the studied systems at 298 K and 1 bar was calculated using a multi‐stage free energy perturbation (FEP) method [74, 75, 76, 77] with the multi‐state Bennett's acceptance ratio (MBAR) method [78, 79, 80, 81] following our previous work [82, 83, 84, 85]. A “soft‐core” potential was used to couple/decouple the paracetamol‐solvent intermolecular LJ interactions. Stage (m) dependent decoupling parameters, $\lambda_{m}^{\mathrm{LJ}}$ and $\lambda_{m}^{\mathrm{elec}}$, controlled the LJ and electrostatic intermolecular interactions, respectively. The decoupling parameters varied from 0 to 1. When $\lambda_{m}^{\mathrm{LJ}}=\lambda_{m}^{\mathrm{elec}}=1$, the solute is fully coupled to the system. When $\lambda_{m}^{\mathrm{LJ}}=\lambda_{m}^{\mathrm{elec}}=0$, the solute is decoupled from the system. The use of a soft‐core potential is important in that while it gives the correct limiting value of the LJ potential, it additionally allows nearly decoupled molecules to overlap with finite energy (and hence finite probability), thereby increasing the phase space overlap between neighboring states. The soft‐core potential had the form [86, 87, 88]

(2) $$U_{\mathrm{LJ}}^{\mathrm{sc}}\left(r_{ij};m\right)=4\lambda_{m}^{\mathrm{LJ}}\varepsilon_{ij}\{\frac{\sigma_{ij}^{12}}{{\left[\left(1-\lambda_{m}^{LJ}\right)\alpha_{LJ}\sigma_{ij}^{6}+r_{ij}^{6}\right]}^{2}}-\frac{\sigma_{ij}^{6}}{\left[\left(1-\lambda_{m}^{LJ}\right)\alpha_{LJ}\sigma_{ij}^{6}+r_{ij}^{6}\right]}\}$$

where $\alpha_{\mathrm{LJ}}$ is a constant, which had a value of 1/2. The electrostatic term in the intermolecular potential was decoupled linearly as

(3) $$U_{\mathrm{elec}}\left(r_{ij};m\right)=\lambda_{m}^{\mathrm{elec}}\frac{1}{4\pi \varepsilon_{0}}\frac{q_{i}q_{j}}{r_{ij}}$$

At each stage m, an independent MD simulation was performed. For each stage m, the total simulation time was 22 ns, with the first 2 ns discarded from analysis as equilibration. The change in the Hamiltonian with the current configuration between stage m and the other stages is computed every 0.20 ps. This is saved for subsequent post‐simulation analysis with MBAR [81] to determine $\Delta G_{2}^{\mathrm{solv}}$. This analysis was performed using the Python implementation of MBAR (PyMBAR) and the GROMACS analysis script distributed with it [89]. The analysis script has implemented an autocorrelation analysis so that only uncorrelated samples are used to determine $\Delta G_{2}^{\mathrm{solv}}$ and the corresponding uncertainty [90, 91, 92].

A total of 15 different stages were used for the free energy calculations, where m=0 corresponds to a noninteracting (ideal gas) state, and m=14 is a fully interacting system. From m=1 to 10 the LJ interactions were increased from $\lambda_{m}^{\mathrm{LJ}}=0.1$ to 1.0 in 10 equal increments of 0.1. Electrostatic interactions were increased in a square root fashion following $\lambda_{m}^{\mathrm{elec}}=\left\{0.50,0.71,0.87,1.00\right\}$ from m=11 to 14. The change in free energy in going from m=0 to 10 (noninteracting to full LJ) we refer to as the LJ contribution, $\Delta G_{2}^{\mathrm{solv},\mathrm{LJ}}$, and the change in free energy in going from m=10 (full LJ with no electrostatic) to 14 (full LJ and full electrostatic) we refer to as the Coulombic contribution, $\Delta G_{2}^{\mathrm{solv},\mathrm{Coul}}$. The total solvation‐free energy is the sum of these two contributions.

The simulation parameters for the free energy calculations were the same as the last step of equilibration except that the equations of motion were integrated with the GROMACS “stochastic dynamics” integrator, corresponding to stochastic or velocity Langevin dynamics integrated with the leap‐frog algorithm [62, 63, 93]. The time constant for the stochastic thermostat was 1.0 ps. This change is necessary as a local thermostat is required to correctly control the temperature of the decoupled and weakly coupled solute molecule. For the systems in which paracetamol is infinitely dilute, single paracetamol molecule in solution is selected to couple/decouple the paracetamol‐solvent intermolecular interactions. For the systems with paracetamol at a finite concentration, a single paracetamol molecule is randomly selected to couple/decouple.

Sample GROMACS input files are provided in the Supporting Information accompanying the electronic version of this article.

### 2.3 Solubility

The equilibrium solubility of paracetamol (or a nonelectrolyte solid solute in general, component 2) in solution is given by the thermodynamic relation:

(4) $$\mu_{2}^{L}\left(T,P,x_{2}\right)-\mu_{2}^{S}\left(T,P\right)=0$$

where T and P are the temperature and pressure, respectively, $\mu_{2}^{L}$ and $\mu_{2}^{S}$ are the chemical potential of the solute in the liquid (L) solution and solid (S) crystalline form, respectively, and $x_{2}$ is mole frac equilibrium solubility. In writing the phase equilibria expression, we make the standard assumption that the solid phase is pure component 2 [94, 95].

Here, we follow the molecular simulation work of Belluci et al. directly [18], who showed that Equation (4) can be simplified to:

(5) $$\ln\;\rho_{2}^{L}\left(T,P,x_{2}\right)+\frac{1}{\mathrm{RT}}\Delta G_{2}^{\mathrm{solv}}\left(T,P,x_{2}\right)-\frac{G_{2}^{S,\mathrm{ex}}\left(T,P\right)}{{\mathrm{RTN}}^{S}}=0$$

where $\rho_{2}^{L}$ is the number density of paracetamol in solution at equilibrium in units of molecules per Å^3^, $\Delta G_{2}^{\mathrm{solv}}/\mathrm{RT}$ is the dimensionless solvation free energy of paracetamol in solution at equilibrium, R is the molar gas constant, $G^{S,\mathrm{ex}}$ is the extensive excess Gibbs free energy of pure crystalline paracetamol, and $N^{S}$ is the total number of paracetamol molecules in the crystalline state. It is important to emphasize that while the other terms are dimensionless, $\rho_{2}^{L}$ has units of molecules per Å^3^, which results from the simplification of the expression by Belluci et al. [18]. Additionally, while the solution phase terms are both composition‐ and solvent‐dependent, the crystalline term is constant and needs only be computed once for a solute of interest. In the present study, we use the value $G_{2}^{S,\mathrm{ex}}/\left(RTN^{S}\right)=-30.54\pm 0.01$ from Belluci et al. [18].

In general, for the application of Equation (5), at solute mole fractions ($x_{2}$) less than the equilibrium solubility, the expression is negative, indicating an affinity for the solution relative to the solid crystalline phase. At equilibrium, the expression is zero. And at solute mole fractions ($x_{2}$) greater than the equilibrium solubility the expression is positive, indicating an affinity for the solid crystalline phase relative to the solution phase. These characteristics naturally present themselves to the use a bracketing method of root finding, which would require a series of sequential simulations. Alternatively, in the present study, we perform a series of simulations in parallel, covering a range of concentrations. We then fit a regression model to the data, which is used to predict the equilibrium solubility. The approach adopted in the present study differs from that of Belluci et al. [18] only in that here we make use of mole fractions ($x_{2}=N_{2}/(N_{1}+N_{2})$) while Belluci et al. used mole ratios ($X_{2}=N_{2}/N_{1}$), where $N_{2}$ and $N_{1}$ are the number of paracetamol and solvent molecules, respectively.

### 2.4 Infinite Dilution Approximation

While the use of Equation (5) is rigorously correct, it requires multiple simulations of the solution phase. Additionally, for poorly soluble systems, very large system sizes may be necessary to access the concentrations of interest. For these cases, we can extrapolate to lower concentrations through application of the “infinite dilution” approximation, which requires only a simulation at the infinite dilution limit ($N_{2}=1$ and $N_{1}\gg N_{2}$). Note that while in this case the mole fraction concentration is greater than the equilibrium solubility, the system is stable, as for the case of $N_{2}=1$ there are no solute–solute interactions. This strategy can also be used to extrapolate to higher concentrations, analogous to using Henry's constant to estimate the solubility of a gas, albeit caution is required.

For this case, we assume that the solute solvation free energy is constant and independent of concentration:

(6) $$\Delta G_{2}^{\mathrm{solv}}\left(T,P,x_{2}\right)\approx \Delta G_{2}^{\mathrm{solv}}\left(T,P,x_{2}\to 0\right)$$

where $x_{2}\to 0$ corresponds to the infinite dilution limit. This results in the updated expression:

(7) $$\ln\;\rho_{2}^{L}\left(T,P,x_{2}\right)+\frac{1}{RT}\Delta G_{2}^{\mathrm{solv}}\left(T,P,x_{2}\to 0\right)-\frac{G^{S,\mathrm{ex}}\left(T,P\right)}{RTN^{S}}=0$$

where we use the infinite dilution solvation free energy and solve for the number density of paracetamol in solution at equilibrium. Assuming the concentration of the simulation is close to the predicted solubility and the infinite dilution approximation is reasonable ($N_{1}\gg N_{2}$), we can estimate the mole fraction solubility from the number density as:

(8) $$x_{2}\approx \frac{V^{\mathrm{tot}}\left(T,P,N_{1},N_{2}=1\right)}{N_{1}}\rho_{2}^{L}$$

where $V^{\mathrm{tot}}$ is the total (extensive) volume for the simulation at infinite dilution, $N_{1}$ is the number of solvent molecules in the simulation at infinite dilution, and $\rho_{2}^{L}$ is the infinite dilution predicted number density at equilibrium. As the predicted solubility increases, the error introduced by Equation (8) increases. We note that this error could be decreased if one could compute the partial molar volume at infinite dilution, but this was not possible from the infinite dilution simulation with the software used in the present study.

## 3 Results and Discussion

### 3.1 Solubility

Plots demonstrating the application of Equation (5) are shown in Figure 2 for ethanol, Figure 3 for DMF, DMSO, and DEAM, and in Figure 4 for acetone, with the predicted solubilities summarized in Table 1. In general for the application of Equation (5), at solute mole fractions less than the equilibrium solubility the expression is negative ($\mu_{2}^{L}<\mu_{2}^{S}$), indicating an affinity for the solution relative to the solid crystalline phase. At equilibrium, the expression is zero ($\mu_{2}^{L}=\mu_{2}^{S}$). And at solute mole fractions greater than the equilibrium solubility the expression is positive, indicating an affinity for the solid crystalline phase relative to the solution phase ($\mu_{2}^{L}>\mu_{2}^{S}$).

**FIGURE 2:**
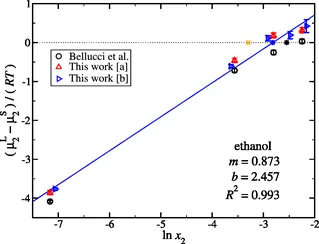
Plot summarizing the calculated values of the phase equilibria expression, Equation (5), in ethanol versus the log mole fraction of paracetamol ($\ln x_{2}$). The black circles correspond to the reference predictions of Belluci et al. [18]. The red triangles, labeled set “a”, correspond to the values directly computed via molecular simulation using the same system sizes and compositions as Belluci et al. The blue triangles, labeled as set “b”, correspond to the values directly computed via molecular simulation following the same procedure as all other solvents in the present study, while the solid blue line corresponds to the linear fit to this data. The blue asterisk corresponds to the equilibrium solubility predicted using the linear fit, while the “x” corresponds to the solubility predicted using the infinite dilution approximation. The predictions are summarized in Table 1.

**FIGURE 3:**
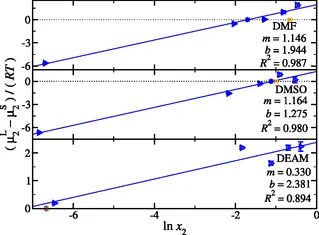
Plot summarizing the calculated values of the phase equilibria expression, Equation (5), versus the log mole fraction of paracetamol ($\ln x_{2}$). The blue triangles correspond to the values directly computed via molecular simulation, while the solid blue line corresponds to the linear fit to this data. The blue asterisk corresponds to the equilibrium solubility predicted using the linear fit, while the “x” corresponds to the solubility predicted using the infinite dilution approximation. The top, middle, and bottom panes correspond to the solvents DMF, DMSO, and DEAM (diethylamine), respectively. The predictions are summarized in Table 1.

**FIGURE 4:**
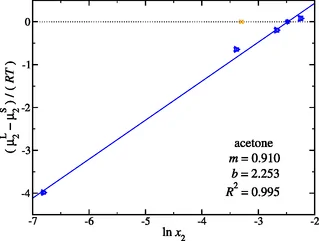
Plot summarizing the calculated values of the phase equilibria expression, Equation (5), in acetone versus the log mole fraction of paracetamol ($\ln x_{2}$). The blue triangles correspond to the values directly computed via molecular simulation, while the solid blue line corresponds to the linear fit to this data. The blue asterisk corresponds to the equilibrium solubility predicted using the linear fit, while the “x” corresponds to the solubility predicted using the infinite dilution approximation. The predictions are summarized in Table 1.

**TABLE 1:** A summary of the simulation predicted and experimental reference equilibrium mole fraction solubility of paracetamol. Molecular simulation predictions are made using Equation (5) (equil) and the infinite dilution approximation (inf) as indicated. The subscript corresponds to the uncertainty in the last reported digit. The experimental data is all from Granberg and Rasmuson [43, 96], except cyclohexane from Barra et al. [44, 97].

**S** **olvent**	**S** **imulation**		**E** **xperimental**
	$x_{2}$ **equil.**	$x_{2}$ **inf.**	$x_{2}$
Ethanol	$6.0_{6}\times 10^{-2}$	$3.7_{2}\times 10^{-2}$	$6.0129\times 10^{-2}$
DEAM	$1_{1}\times 10^{-3}$	$1.29_{4}\times 10^{-3}$	$3.8920\times 10^{-1}$ a
DMSO	$3.3_{7}\times 10^{-1}$	$8.3_{5}\times 10^{-1}$	$3.6927\times 10^{-1}$ a
Acetone	$8_{2}\times 10^{-2}$	$5.9_{2}\times 10^{-2}$	$3.6941\times 10^{-2}$
DMF	$1.8_{3}\times 10^{-1}$	$3.5_{2}\times 10^{-1}$	$3.2856\times 10^{-1}$ a
Cyclohexane		$3.7_{1}\times 10^{-5}$	$2.000\times 10^{-5}$
Toluene		$1.67_{6}\times 10^{-3}$	$2.2548\times 10^{-4}$
Water		$1.42_{8}\times 10^{-4}$	$1.7726\times 10^{-3}$

a Reference data is at 303.15 K.

We first consider the case of ethanol. In Figure 2, three sets of predictions are presented. First, we have the reference predictions from the work of Belluci et al. [18]. Next, for our first set of predictions labeled “a”, we use the exact system sizes of Belluci et al. but slightly different simulation parameters and protocol adopted in the present study. This includes different versions of GROMACS (5.1.4 vs. 2020.2) and methodologies used to predict the solvation free energy (the FEP implementation available in GROMACS 5.1.4 vs. the use of MBAR using the external software PyMBAR). We find that in the present study, we consistently predict a solute liquid‐phase chemical potential that is slightly greater than that of Belluci et al. Subsequently, our second set of predictions is labeled “b”. This set makes use of the simulation parameters and protocol adopted in the present study, most noticeably in the determination of the system size (target a cubic box with an edge length of approximately 4.8 nm) and the concentrations studied (infinite dilution plus three or four additional concentrations in equal increments from infinite dilution to beyond the experimental solubility). With this set we predict an equilibrium solubility of 0.060±0.006 mole fracs, in excellent agreement with the experimental value of 0.060129 mole fracs [43, 96]. This also agrees well with and is statistically equivalent to the equilibrium solubility prediction of Belluci et al. [18] of 0.0585±0.004 mole ratios or 0.055±0.004 mole fracs. This agreement gives us confidence as we move forward with additional solvents.

Likewise, we find that the equilibrium solubility predicted for paracetamol in DMSO, acetone, and DMF is in good agreement with the experiment. Interestingly, we find that for ethanol and acetone, the infinite dilution prediction is less than that predicted using Equation (5), while for DMSO and DMF, the infinite dilution prediction is greater than that predicted using Equation (5) . While the direction of the difference is not consistent, we do find here that the infinite dilution predictions are of the same order of magnitude and could be used to provide a reasonable estimate of the equilibrium solubility. In the absence of experimental data, this could be valuable in guiding the system sizes selected and an initial estimate of whether the simulation conditions are reasonable.

Knowledge of the sign of Equation (5) is invaluable for understanding the state of the system. Plots are not provided for the case of cyclohexane, toluene, and water where the experimental solubility is less than $1\times 10^{-3}$ mole fracs. For all of these cases except toluene, the molecular simulations at infinite dilution, for which we had a cubic box edge of approximately 4.8 nm, $\mu_{2}^{L}<\mu_{2}^{S}$ indicating the concentration was beyond the equilibrium solubility. This was additionally the case for diethylamine (DEAM), as depicted in the bottom plot of Figure 3. For DEAM, simulations were performed at a series of higher concentrations because the reported experimental equilibrium solubility at 303.15 K is 0.3892 mol fracs [44, 97]. Given the reasonable agreement for ethanol, DMSO, acetone, and DMF, and considering the systems with the highest solubilities for which simulations were performed at concentrations beyond the infinite dilution limit, the macroscopic solubility predictions indicate a clear limitation of the model for the DEAM system. Subsequently, in the “Solution Structure” subsection below, we perform a detailed microscopic structural analysis to study the evolution of the solution structure as a function of concentration in an effort to pinpoint these model deficiencies. This limitation has two important implications, highlighting the value of Equation (5) and the modeling of solubility. (1) There may exist a need to model a solution at or near saturation, for example, in the study of nucleation. Here, the application of Equation (5) even at saturation would reveal the poor performance of the model in reproducing the experimental phase equilibrium. Furthermore, simulations conducted at concentrations greater than the equilibrium solubility predicted by the model would correspond to a metastable state and would not be representative of the system of interest. While the system sizes and timescales are relatively small so that phase separation would not be observed, they would nonetheless correspond to a metastable system. We emphasize that what is important is the equilibrium solubility of the model, which may differ from the experiment. (2) This provides a route wherein phase equilibrium may be incorporated into the parameterization of biological systems of interest, analogous, for example, to the parameterization of small molecules using VLE [34, 35].

For the case of cyclohexane, DEAM, and water, at the infinite dilution ($N_{2}=1$ and $N_{1}\gg N_{2}$) limit considered in the present study, we found that Equation (5) is greater than 0, indicating that we are at a concentration greater than the equilibrium solubility of the model. Note that while in this case the mole fraction concentration is greater than the equilibrium solubility, the system is stable, as for the case of $N_{2}=1$ there are no solute–solute interactions. For these cases, we extrapolate to lower concentration through application of the infinite dilution approximation. On the other hand, for the case of ethanol, DMSO, acetone, and DMF, the infinite dilution approximation was used to extrapolate from the infinite dilution limit to higher concentrations, analogous to the use of Henry's law in the modeling of the solubility of noncondensable gases.

As noted, for the case of toluene, the simulation at the infinite dilution ($N_{2}=1$ and $N_{1}=627$) limit considered in the present study corresponded to a mole fraction of $x_{2}=1.592\times 10^{-3}$ mole fracs and we obtain a value of Equation (5) of −0.0436. While the negative value indicates that we are below the equilibrium solubility, the small value tells us that we are close. The result of using the infinite dilution approximation is very close and $1.67\times 10^{-3}$ mole fracs.

In the Supporting Information accompanying the electronic version of this article, we provide a spreadsheet tabulating the simulation results and system sizes. This includes the results of the free‐energy calculations with uncertainties.

#### 3.1.1 Infinite Dilution Approximation

Let us next investigate the physical and thermodynamic underpinnings of the infinite dilution approximation prior to discussing the detailed structural analysis. In the present study, we have shown that the solubility predictions obtained via the infinite dilution approximation are of the same order of magnitude as the rigorous concentration‐dependent equilibrium solubility predictions. Consequently, this approximation can serve as a highly reasonable first‐order estimate of equilibrium solubility when full free‐energy curves or experimental data are unavailable.

Interestingly, we find that for ethanol and acetone, the infinite dilution approximation underestimates the rigorous equilibrium solubility, whereas the opposite trend is observed for DMSO and DMF. To understand this departure of the solvation free energy from the infinite dilution limit as a function of concentration, we define the free‐energy difference as:

(9) $$\Delta \Delta G_{2}^{\mathrm{solv}}\left(T,P,x_{2}\right)=\Delta G_{2}^{\mathrm{solv}}\left(T,P,x_{2}\right)-\Delta G_{2}^{\mathrm{solv}}\left(T,P,x_{2}\to 0\right)$$

In Figure 5, we plot the calculated dimensionless values of $\Delta \Delta G_{2}^{\mathrm{solv}}/\left(\mathrm{RT}\right)$ versus the logarithm of the paracetamol mole fraction ($\ln\;x_{2}$). First, we observe that for DMSO and DMF, the value of $\Delta G_{2}^{\mathrm{solv}}$ increases (i.e., becomes less negative and thermodynamically less favorable) with increasing paracetamol concentration. Qualitatively, this behavior agrees with the “dilute solution” approximation previously proposed and applied in solubility predictions [21, 22, 23]. Under this framework, solute–solute interactions are assumed to be negligible, and explicit free‐energy calculations are performed only at the infinite dilution limit. Rather than assuming that the solvation free energy remains strictly constant, the deviation relative to the infinite dilution limit is modeled using an athermal (or combinatorial) excess Gibbs free energy model, such as the Flory–Huggins equation.

**FIGURE 5:**
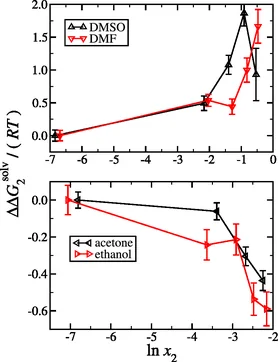
Plot summarizing the calculated dimensionless values of $\Delta \Delta G_{2}^{\mathrm{solv}}/\left(\mathrm{RT}\right)$ (see Equation (9)) versus the logarithm of the mole fraction of paracetamol ($\ln x_{2}$). The top panel contains results for DMSO and DMF, and the bottom panel contains results for acetone and ethanol.

Recall that the solvation free energy corresponds to the change in Gibbs free energy upon transferring a solute from a noninteracting ideal gas phase to the solution at the same molar concentration (molecules or moles per volume) [98]. For DMSO and DMF, the solvation of paracetamol becomes progressively less favorable as the paracetamol mole fraction increases, representing typical crowding and volume‐exclusion effects. On the other hand, for acetone and ethanol, $\Delta G_{2}^{\mathrm{solv}}$ decreases (becomes more negative and thermodynamically more favorable) with increasing paracetamol mole fraction. In these systems, paracetamol solvation becomes more favorable as its concentration increases, which is opposite to the behavior in DMSO and DMF. This result stands in stark contrast to the dilute solution approximation, indicating that such approximations must be applied with caution in systems where specific, attractive solute–solute, or cooperative solvation interactions are present.

The mathematical origin of these over‐ and underestimations becomes clear when examining the overall driving force for dissolution, $\mu_{2}^{L}-\mu_{2}^{S}$. In Figures 2–4, we observed that $\mu_{2}^{L}-\mu_{2}^{S}$ increases monotonically with the mole fraction of paracetamol, where the solid‐phase chemical potential $\mu_{2}^{S}$ is constant. The liquid‐phase chemical potential $\mu_{2}^{L}$ is composed of the solvation free energy ($\Delta G_{2}^{\mathrm{solv}}$) plus the logarithm of the paracetamol number density in solution ($\ln\;\rho_{2}^{L}$) (see Equation (5)). As the mole fraction of paracetamol increases, the density term $\ln\;\rho_{2}^{L}$ naturally increases.

In acetone and ethanol, because $\Delta G_{2}^{\mathrm{solv}}$ decreases (becomes more favorable) with concentration, the actual rate of increase of $\mu_{2}^{L}-\mu_{2}^{S}$ is slower than it would be if $\Delta G_{2}^{\mathrm{solv}}$ remained constant. Consequently, the infinite dilution approximation (which holds $\Delta G_{2}^{\mathrm{solv}}$ constant at its infinite dilution value) overestimates the rate of increase of $\mu_{2}^{L}-\mu_{2}^{S}$. This overestimation of the slope causes the approximated chemical potential to intersect the solid chemical potential limit at a lower concentration, resulting in a solubility prediction that is lower than the rigorous equilibrium solubility prediction. Conversely, in DMSO and DMF, where $\Delta G_{2}^{\mathrm{solv}}$ increases with concentration, the infinite dilution approximation underestimates the rate of increase of $\mu_{2}^{L}-\mu_{2}^{S}$ because it neglects this additional positive free energy contribution. This underestimation of the slope causes the chemical potential curve to climb too slowly, intersecting the solid limit at a higher concentration and thus yielding an overestimated solubility prediction.

Furthermore, by directly comparing the components of $\mu_{2}^{L}-\mu_{2}^{S}$, we can gain deep physical insight into why the infinite dilution approximation still yields remarkably reasonable first‐order predictions. Specifically, the concentration‐dependent change in the number density term, $\ln\;\rho_{2}^{L}$, is significantly larger than the corresponding change in the solvation free energy, $\Delta G_{2}^{\mathrm{solv}}$. For the case of DMSO, at the highest concentration of $x_{2}=0.403$, we find that relative to the infinite dilution limit ($N_{2}=1$), the dimensionless solvation contribution $\Delta G_{2}^{\mathrm{solv}}/\left(\mathrm{RT}\right)$ increases by 1.9±0.2, while the density term $\ln\;\rho_{2}^{L}$ increases by a dominant 5.6678±0.0003. Similarly, for the case of ethanol at its highest concentration, $\Delta G_{2}^{\mathrm{solv}}/\left(\mathrm{RT}\right)$ decreases by −0.59±0.09 relative to the infinite dilution limit ($N_{2}=1$), whereas $\ln\;\rho_{2}^{L}$ increases by 4.7788±0.0002.

The much larger magnitude of the change in $\ln\;\rho_{2}^{L}$ relative to the variation in $\Delta G_{2}^{\mathrm{solv}}$ explains the overall robust performance of the infinite dilution approximation. Furthermore, the smaller absolute shift in $\Delta G_{2}^{\mathrm{solv}}$ for ethanol (−0.59) compared to DMSO (+1.9) explains why the infinite dilution approximation is quantitatively more accurate (on an absolute mole fraction basis) in ethanol than in DMSO. Similar consistent trends are observed for DMF and acetone.

### 3.2 Solution Structure

For water and the organic solvents, we considered the case of a single paracetamol molecule ($N_{2}=1$) infinitely dilute in solution, corresponding to the lowest mole fraction composition. For ethanol, DMSO, DMF, diethylamine, and acetone, simulations were performed at three or four additional concentrations of paracetamol. These concentrations were increased in equal increments of $N_{2}$ from infinite dilution to beyond the experimental solubility. After comparing the equilibrium solubility predicted by the molecular models to experimental data (summarized in Table 1), we next consider the underlying simulation structure as a function of composition. Here, we do not examine the solution structure of paracetamol in cyclohexane, toluene, and water because simulations were performed only at the infinite dilution limit. When molecular simulations were performed at mole fraction compositions greater than the equilibrium solubility predicted by the molecular models, it corresponds to a metastable system. Given the relatively small system sizes and timescales in the simulations, coupled with the use of periodic boundary conditions, we would not expect crystallization to be observed. Nucleation is a rare event, and observing a liquid‐to‐solid phase transition is unrealistic. Nonetheless, this would correspond to a metastable system.

#### 3.2.1 Solution Heterogeneity

A snapshot from our equilibrium molecular simulation for paracetamol in DMSO at each concentration studied is provided in Figure 6. Green is used to represent paracetamol molecules, and rust is used to represent DMSO molecules to visualize the homogeneous or inhomogeneous structure of the system. As a reference, in DMSO, the model predicts an equilibrium mole fraction solubility of $3.259\times 10^{-1}$, which would fall between our simulations at *N*_2_ = 200 and 300. The images suggest that we maintain a homogeneous structure through the highest concentration considered, $x_{2}=0.587$ ($N_{2}=400$). This highlights the challenge of using structural features as a signature of equilibrium solubility.

**FIGURE 6:**
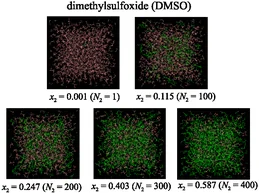
A snapshot of the central simulation cell for paracetamol in DMSO at each concentration studied, as indicated. Green and rust are used to represent paracetamol and DMSO molecules, respectively, and $x_{2}$ and $N_{2}$ correspond to the mole fraction and number of paracetamol molecules, respectively.

Interestingly, in the middle pane of Figure 3, there appears to be an abrupt change in the linear trend of the solubility expression versus the log mole fraction between *x*_2_ = 0.403 and 0.587 (*N*_2_ = 300 and 400), just above the equilibrium solubility predicted by the model. This is similar to previous work wherein molecular simulation was used to compute the pure liquid fugacity of paracetamol [82]. While the freezing (or melting) point of the molecular model was unknown, the linear Clapeyron plot of the log fugacity versus inverse temperature exhibited an abrupt change in slope at a temperature just below the experimental freezing point. This would correspond to a step change in the enthalpy of vaporization and the presence of a first‐order phase transition. As already noted, nucleation is a rare event, and observing a liquid‐to‐solid phase transition is unrealistic. This comparison is interesting, wherein one is not able to observe a phase transition from a structural perspective. Still, the underlying free energy calculation suggests a thermodynamic signature that a phase transition has occurred, albeit not in perfect agreement with rigorous phase equilibrium calculations. We note that this abrupt change in the linear trend is not limited to DMSO but appears in the other solvents and the reference work of Belluci et al. [18].

Next, in Figure 7 we have a snapshot from our equilibrium molecular simulation for paracetamol in diethylamine at each concentration studied. For this case, the molecular models estimate a mole fraction solubility of approximately $1.274\times 10^{-3}$ at 298.15 K, whereas the experimental value at 303.15 K is two orders of magnitude higher at $3.892\times 10^{-1}$ [43, 96]. As a result, all but the first molecular simulation at infinite dilution ($N_{2}=1$) are at a concentration greater than the predicted solubility. From Figure 7, we find that at $N_{2}=100$, the system is inhomogeneous with the formation of well‐defined aggregates or clusters of diethylamine. If one were to model the system at the experimental equilibrium solubility concentration, they would obtain an inhomogeneous system that would not be representative of the equilibrium system.

**FIGURE 7:**
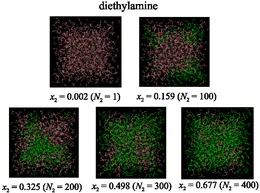
A snapshot of the central simulation cell for paracetamol in diethylamine at each concentration studied, as indicated. Green and rust are used to represent paracetamol and diethylamine molecules, respectively, and $x_{2}$ and $N_{2}$ correspond to the mole fraction and number of paracetamol molecules, respectively.

The formation of stable clusters has recently been proposed as a rapid diagnostic for the onset of phase transitions in simple electrolytes [26]. However, considering the case of DMSO and DEAM, our results indicate that such structural features are not a quantitatively accurate universal indicator of thermodynamic equilibrium for complex molecular solutes.

The snapshots from our equilibrium molecular simulations for paracetamol qualitatively show that for the case of DMSO, the system remains homogeneous, whereas for the case of diethylamine, inhomogeneities exist. We next quantitatively confirm this behavior using spatial distribution and local density fluctuations of paracetamol in solution, specifically the microheterogeneity analysis method of Brehm and coworkers [99] as implemented in Travis [100, 101]. Unlike conventional cluster‐size analyses that rely on arbitrary, sharp distance cutoffs to define molecular domains, this method characterizes the liquid structure via spatial density fluctuations across continuous length scales. We will provide a brief description of the method here, but the original article should be consulted for further details and derivation [99].

To perform this analysis, a three‐dimensional Cartesian grid (comprising 35×35×35 sampling points) was projected over the periodic simulation cell. At each grid point, a virtual sampling sphere of radius $r_{S}$ (swept from 100 to 2400 pm in steps of 50 pm) was placed, and the local number density of paracetamol was computed by tracking the coordinates of its molecular center of mass. By scanning all grid positions across the 100 ns trajectory, a probability distribution of the local solute density was constructed for each sphere radius.

The standard deviation of these observed local densities ($\sigma_{\text{Observed}}$) was compared to the analytical binomial standard deviation of an ideal gas ($\sigma_{\text{IdealGas}}$) at the identical overall density to calculate the relative heterogeneity index, defined as $h_{\text{rel}}=\sigma_{\text{Observed}}/\sigma_{\text{IdealGas}}$. Values of $h_{\text{rel}}<1.0$ indicate a highly uniform, regularly spaced distribution of solute molecules (stabilized by volume exclusion and solvation), while $h_{\text{rel}}>1.0$ quantitatively proves unphysical spatial fluctuations and domain clustering. Concurrently, the empty sphere probability was evaluated as the probability of a randomly placed sampling sphere of radius $r_{S}$ containing zero solute centers of mass, providing a direct measurement of the characteristic dimensions of solvent‐rich domains. When considering these results, recall that the simulation system sizes ($N_{1}$ and $N_{2}$) of the present study were determined to achieve a target cubic box with an edge length of approximately 4.8 nm (or 4800 pm); the corresponding concentrations in DMSO and diethylamine are therefore at approximately the same density (molar or mass).

First, plots of the probability of finding an empty sampling sphere as a function of sphere radius for paracetamol in DMSO and diethylamine at each concentration studied are provided in Figure 8. In DMSO, the empty sphere probability of paracetamol drops off quickly. For the case of the highest concentration, $N_{2}=400$, it abruptly goes to zero at a radius of 550 pm. Even at the lowest concentration, $N_{2}=100$, the empty sphere probability goes to zero by 1150 pm. The latter case illustrates that paracetamol is well dispersed such that there are no paracetamol‐free spheres larger than 1150 pm (or 1.15 nm) in the system. On the other hand, in diethylamine, the decay is dramatically slower, corresponding to a relatively larger population of larger solvent‐only spheres. For the case of $N_{2}=400$, the empty sphere probability of paracetamol does not drop to zero until 1100 pm, double the distance for the corresponding case in DMSO. Moreover, for $N_{2}=100$, the empty sphere probability tails out to large distances, finally hitting zero at 2200 pm (or 2.2 nm). This result is aligned with the clustering of paracetamol in diethylamine as observed in Figure 7.

**FIGURE 8:**
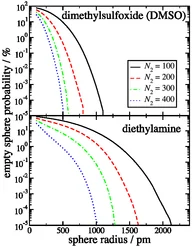
Plot of the probability of finding an empty sampling sphere as a function of sphere radius for paracetamol in DMSO (top pane) and diethylamine (bottom pane) at each concentration studied ($N_{2}>1$, see Figures 6 and 7).

Next, this microphase separation and domain formation are further quantified and validated by the relative heterogeneity curves ($h_{\text{rel}}$) shown in Figure 9. In the homogeneous DMSO system, the relative heterogeneity curves for all concentrations display a local packing peak at 300 pm (reaching 0.74 for $N_{2}=400$), which corresponds to the first solvation shell of the paracetamol monomers. Beyond this local shell, $h_{\text{rel}}$ steadily decays to a flat, highly suppressed plateau at larger length scales. Further, the value of this large‐scale plateau decreases systematically with increasing solute concentration, dropping from 0.65 at $N_{2}=100$ to 0.33 at $N_{2}=400$ at a radius of 2400 pm. This behavior agrees with the thermodynamic signature of a stable, homogeneous crowded phase; as the density of uniformly dispersed paracetamol monomers increases, volume exclusion forces them into a highly regular spatial arrangement, suppressing density fluctuations far below those of a random ideal gas ($h_{\text{rel}}\ll 1.0$) [99].

**FIGURE 9:**
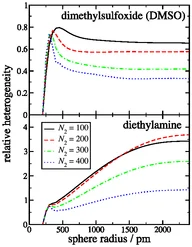
Plot of the relative heterogeneity index ($h_{\text{rel}}$) as a function of sphere radius for paracetamol in DMSO (top pane) and diethylamine (bottom pane) at each concentration studied ($N_{2}>1$, see Figures 6 and 7).

In stark contrast, the relative heterogeneity in diethylamine exhibits a complete reversal of this behavior, demonstrating severe, unphysical solute clustering across all finite concentrations. While the curves still exhibit a brief local packing peak at 300 pm, they do not decay at larger length scales. For the highest concentration ($N_{2}=400$), $h_{\text{rel}}$ hits a minimum at 400 pm but then climbs steadily, crossing the random ideal gas threshold ($h_{\text{rel}}=1$) at 1100 pm, the radius where the empty‐sphere probability for $N_{2}=400$ drops to zero.

A particularly telling trend is observed in the concentration dependence of the large‐scale heterogeneity in DEAM. Unlike DMSO, where crowding suppresses fluctuations, the large‐scale heterogeneity at $r_{S}=2400$ pm in diethylamine increases dramatically as concentration decreases, rising from 1.42 at $N_{2}=400$ to a value of 3.42 at $N_{2}=100$. This trend reflects the extreme spatial asymmetry of the phase‐separated system: at low concentration ($N_{2}=100$), the paracetamol molecules aggregate into a single, highly localized cluster, leaving behind an enormous, pure‐solvent void that dominates the volume of the simulation box. This extreme density contrast between the cluster and the vacant solvent domain drives massive spatial fluctuations on large‐length scales. As the solute concentration increases to $N_{2}=400$, the clusters occupy a larger fraction of the simulation cell, capping the maximum solvent void size at approximately 1100 pm (as shown in Figure 8) and moderately reducing the amplitude of the density fluctuations at the box boundary, though the system remains highly heterogeneous ($h_{\text{rel}}\gg 1.0$). Taken together, these multiscale spatial fluctuation metrics provide thermodynamic‐based proof of unphysical phase separation driven by force field imbalances in the diethylamine model [99].

The Supporting Information accompanying the electronic version of this article contains equivalent figures for acetone, DMF, and ethanol (Figures S1–S5).

#### 3.2.2 Atomistic Interactions

All of the subsequent structural analysis was performed with Travis [100, 101]. The atomic labels used through the analysis presented here are provided in Figure 10. We consider first the use of connection matrix (CMat) analysis in Figures 11–13 for DMSO, diethylamine, and ethanol, respectively [101]. In all cases, the rows in the matrix correspond to hydrogen‐bond‐accepting sites and the columns correspond to hydrogen‐bond‐donating sites. The colors represent the intensity and distance of the first maximum in the local density, $\rho_{i,j}\left(r_{i,j}\right)$ (i.e., the un‐normalized RDF), as indicated by the scale on the right. The presence of a black cross indicates that no interaction was detected within a distance of 350 pm.

**FIGURE 10:**
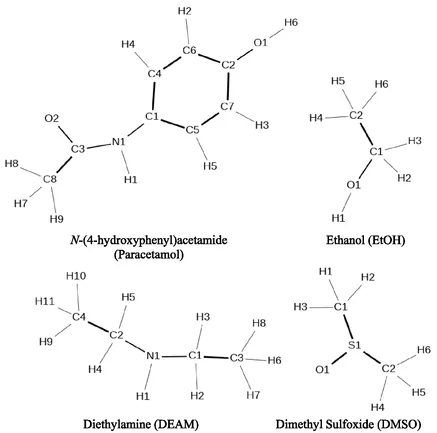
Chemical structure of paracetamol, ethanol (EtOH), diethylamine (DEAM), and dimethyl sulfoxide (DMSO) and the labeling scheme used when performing structural analysis.

Considering first the case of DMSO in Figure 11, at infinite dilution ($N_{2}=1$) we find the dominant interaction is between H6 of paracetamol, corresponding to the hydroxyl hydrogen, and O1 of DMSO. This is followed closely by the H1 of paracetamol, corresponding to the amide hydrogen and O1 of DMSO. In all cases, paracetamol is the hydrogen‐bond donor and DMSO is the hydrogen‐bond acceptor. This is as expected, where DMSO is a strong hydrogen‐bond acceptor but is not capable of donating a hydrogen bond. In addition, a hydroxyl group (OH) is a stronger hydrogen‐bond donor/acceptor as compared to the NH of the amide group [102]. These remain the predominant interactions through $N_{2}=400$, past the equilibrium solubility. Paracetamol···paracetamol interactions do exist, wherein the hydroxyl (O1) or amide (O2) oxygen is the hydrogen bond acceptor, and the hydroxyl (H6) or amide (H1) hydrogen is the hydrogen bond donor. However, DMSO is the predominant hydrogen‐bond acceptor.

**FIGURE 11:**
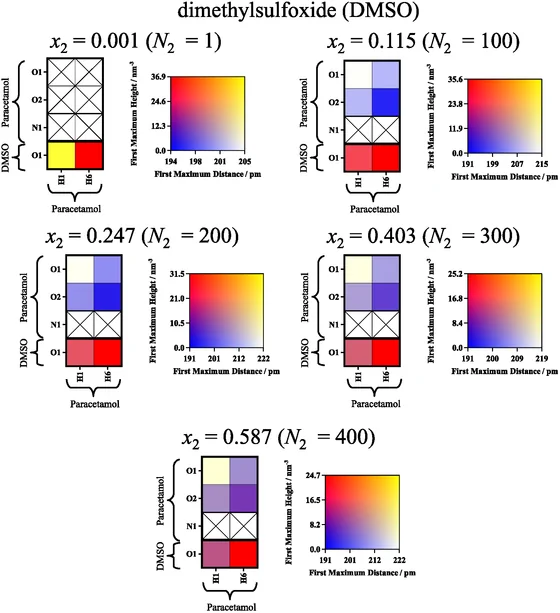
Connection matrix analysis for paracetamol in dimethyl sulfoxide (DMSO) for each concentration studied. The rows represent hydrogen bond acceptors, and the columns represent hydrogen bond donors. The colors represent the intensity and distance of the first maximum in the local density, $\rho_{i,j}(r_{i,j})$ (i.e., the un‐normalized radial distribution function), as indicated in the scale on the right, and $x_{2}$ and $N_{2}$ correspond to the mole fraction and number of paracetamol molecules, respectively. The presence of a black cross indicates no interaction was detected within a distance of 350 pm.

Continuing our discussion of paracetamol···DMSO interactions, Figure 14 contains the RDF analysis, and Figure 15 contains the corresponding number integral analysis. From the RDF analysis, we find that the solution structure does not change with respect to the concentration of paracetamol, even past the equilibrium solubility. We do find that for H1···O1 the intensity of the first maximum changes little going from $N_{2}=1$ to $N_{2}=400$ (3.435 to 3.417), while for H6···O1 there is a noticeable increase (4.381 to 7.171). The increase in the H6···O1 intensity suggests that the strength of the H6···O1 hydrogen bond increases with increasing paracetamol concentration. From the number integral analysis, Figure 15, we find that at infinite dilution ($N_{2}=1$), the paracetamol hydroxyl (H6) and amide (H1) hydrogens are coordinated with a DMSO molecule. As the mole fraction of paracetamol increases (and the mole fraction of DMSO decreases), the coordination number decreases.

We next consider the case of diethylamine. From the CMat analysis in Figure 12, at infinite dilution ($N_{2}=1$), the dominant interaction is between diethylamine and paracetamol, where the amino hydrogen (H1) of diethylamine is the donor, and paracetamol is the hydrogen acceptor with the amide oxygen (O2) followed closely by the hydroxyl oxygen (O1). However, beyond the infinite dilution limit, this is overcome by paracetamol···paracetamol hydrogen bonding. The dominant paracetamol···paracetamol interaction is between the hydroxyl hydrogen (H6) and the amide oxygen (O2). This is followed closely by the amide hydrogen (H1) with the amide oxygen (O2) and the hydroxyl hydrogen (H6) with the hydroxyl oxygen (O1). With the molecular models for diethylamine and paracetamol, we predicted an equilibrium mole fraction solubility of paracetamol of approximately $1.274\times 10^{-3}$ at 298.15 K as compared to a reference value of $3.892\times 10^{-1}$ at 303.15 K [43, 96]. Comparing the case of diethylamine to DMSO, we greatly under‐predict the equilibrium solubility in diethylamine as a result of the molecular model under‐predicting the hydrogen‐bonding strength between diethylamine and paracetamol.

**FIGURE 12:**
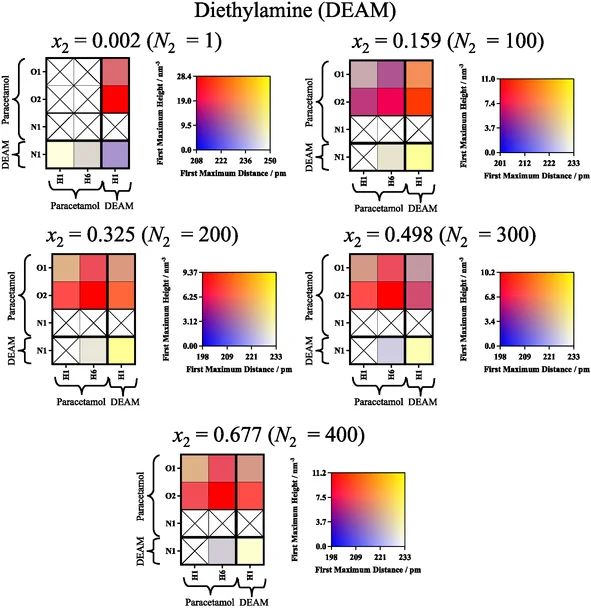
Connection matrix analysis for paracetamol in diethylamine (DEAM) for each concentration studied. The rows represent hydrogen bond acceptors, and the columns represent hydrogen bond donors. The colors represent the intensity and distance of the first maximum in the local density, $\rho_{i,j}(r_{i,j})$ (i.e., the un‐normalized radial distribution function), as indicated in the scale on the right, and $x_{2}$ and $N_{2}$ correspond to the mole fraction and number of paracetamol molecules, respectively. The presence of a black cross indicates no interaction was detected within a distance of 350 pm.

In Figures 16 and 17, we consider the RDF analysis and the corresponding number integral analysis, respectively, which further confirm that the models greatly under‐predict the hydrogen‐bonding strength between diethylamine and paracetamol. Considering paracetamol···diethylamine, we see a drastic decrease in the first peak intensity in going from $N_{2}=1$ to $N_{2}=100$. This is likewise reflected in the number‐integral analysis. Next, in the RDF analysis of diethylamine (H1)···diethylamine (N1) we observe a first peak intensity near one, and a low albeit higher second broad peak, suggesting an absence of H1···N1 hydrogen bonding.

We next consider ethanol as a comparison to diethylamine. Like diethylamine, ethanol is both a hydrogen‐bond donor and acceptor. From the CMat analysis in Figure 13, the dominant interaction is between ethanol as the hydrogen bond donor (H1), with the hydroxyl group oxygen (O1) of ethanol and the amide group oxygen (O2) of paracetamol as the hydrogen bond acceptor, followed closely by the hydroxyl group oxygen (O1) of paracetamol. As compared to diethylamine, this trend remains at all concentrations considered, and this includes solvent···solvent hydrogen bonding. We acknowledge that the range of concentrations considered is much smaller for ethanol as compared to that for diethylamine. The reason for this is that the experimental mole fraction solubility of paracetamol in ethanol is an order of magnitude smaller than in diethylamine. Despite this, interestingly, the equilibrium mole fraction solubility predicted by the molecular models is an order of magnitude larger in ethanol than in diethylamine, and moreover, the predicted value in ethanol is in good agreement with the experiment. We can reasonably compare the highest concentration of paracetamol in ethanol ($x_{2}=0.115$ or $N_{2}=120$) to the second lowest concentration in diethylamine ($x_{2}=0.159$ or $N_{2}=100$), and clearly find that the models underestimate the strength of hydrogen bonding between paracetamol and diethylamine, and between diethylamine with itself.

**FIGURE 13:**
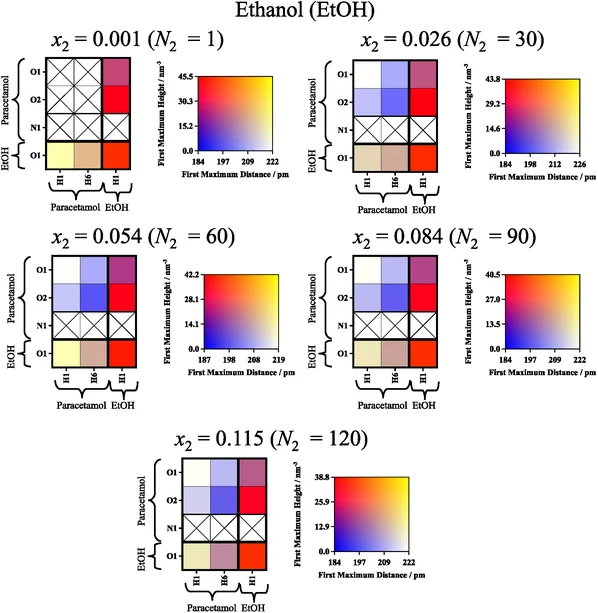
Connection matrix analysis for paracetamol in ethanol (EtOH) for each concentration studied. The rows represent hydrogen bond acceptors, and the columns represent hydrogen bond donors. The colors represent the intensity and distance of the first maximum in the local density, $\rho_{i,j}(r_{i,j})$ (i.e., the un‐normalized radial distribution function), as indicated in the scale on the right, and $x_{2}$ and $N_{2}$ correspond to the mole fraction and number of paracetamol molecules, respectively. The presence of a black cross indicates no interaction was detected within a distance of 350 pm.

**FIGURE 14:**
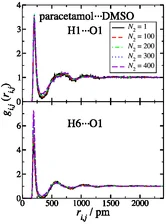
Radial distribution function analysis, $g_{i,j}$, for paracetamol···dimethyl sulfoxide (DMSO) interactions. The top pane contains H1 (paracetamol)···O1 (DMSO) and the bottom pane contains H6 (paracetamol)···O1 (DMSO) interactions. $N_{2}$ corresponds to the number of molecules of paracetamol in the simulation, which ranges from $x_{2}=0.001$ to $x_{2}=0.587$. For H1···O1, the intensity of the first maximum changes little, going from $N_{2}=1$ to $N_{2}=400$ (3.435 to 3.417), while for H6···O1 there is a noticeable increase (4.381 to 7.171).

**FIGURE 15:**
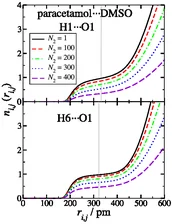
Number integral analysis, $n_{i,j}$, for paracetamol···dimethyl sulfoxide (DMSO) interactions, complementary to Figure 14. The top pane contains H1 (paracetamol)···O1 (DMSO) and the bottom pane contains H6 (paracetamol)···O1 (DMSO) interactions. $N_{2}$ corresponds to the number of molecules of paracetamol in the simulation, which ranges from $x_{2}=0.001$ to $x_{2}=0.587$. The vertical gray line is drawn as a reference and corresponds to the location of the first minimum (i.e., the first solvation shell) for $N_{2}=400$ from Figure 14.

**FIGURE 16:**
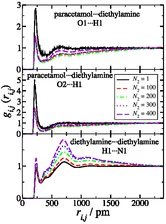
Radial distribution function analysis, $g_{i,j}$, for paracetamol···diethylamine (DEAM) and DEAM···DEAM interactions. The top pane contains O1 (paracetamol)···H1 (DEAM), the middle pane contains O2 (paracetamol)···H1 (DEAM), and the bottom pane contains H1 (DEAM)···N1 (DEAM) interactions. $N_{2}$ corresponds to the number of molecules of paracetamol in the simulation, which ranges from $x_{2}=0.002$ to $x_{2}=0.677$. For paracetamol···DEAM the intensity of the first maximum decreases going from $N_{2}=1$ to $N_{2}=400$ (2.851 to 1.539 for O1···H1, and 5.035–2.367 for O2···H1). For DEAM···DEAM the intensity of the first maximum increases slightly (1.128 to 1.811) going from $N_{2}=1$ to $N_{2}=400$.

**FIGURE 17:**
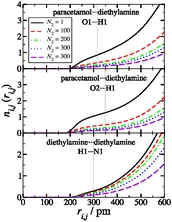
Number integral analysis, $n_{i,j}$, for paracetamol···diethylamine (DEAM) and DEAM···DEAM interactions, complementary to Figure 16. The top pane contains O1 (paracetamol)···H1 (DEAM), the middle pane contains O2 (paracetamol)···H1 (DEAM), and the bottom pane contains H1 (DEAM)···N1 (DEAM) interactions. $N_{2}$ corresponds to the number of molecules of paracetamol in the simulation, which ranges from $x_{2}=0.002$ to $x_{2}=0.677$. The vertical gray line is drawn as a reference and corresponds to the location of the first minimum (i.e., the first solvation shell) for $N_{2}=400$ from Figure 16.

In Figures 18 and 19, we consider the RDF analysis and the corresponding number integral analysis, respectively, for paracetamol and ethanol. Considering the dominant interactions, the RDF analysis shows that the solvation structure is unchanged with respect to the concentration. Moreover, the first peak intensity changes only slightly. This is reinforced by the number integral analysis, where we see only a slight decrease with increasing paracetamol concentration.

**FIGURE 18:**
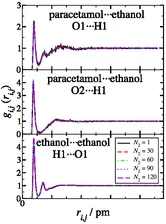
Radial distribution function analysis, $g_{i,j}$, for paracetamol···ethanol and ethanol···ethanol interactions. The top pane contains O1 (paracetamol)···H1 (ethanol), the middle pane contains O2 (paracetamol) ···H1 (ethanol), and the bottom pane contains H1 (ethanol)···O1 (ethanol) interactions. $N_{2}$ corresponds to the number of molecules of paracetamol in the simulation, which ranges from $x_{2}=0.001$ to $x_{2}=0.115$. For paracetamol···ethanol, the intensity of the first maximum decreases very slightly, going from $N_{2}=1$ to $N_{2}=120$ for O1···H1 (2.270 to 2.134), and increases very slightly for O2···H1 (3.908 to 4.100). For ethanol···ethanol the intensity of the first maximum increases slightly (4.324 to 4.744) going from $N_{2}=1$ to $N_{2}=120$.

**FIGURE 19:**
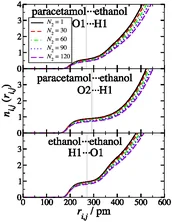
Number integral analysis, $n_{i,j}$, for paracetamol···ethanol and ethanol···ethanol interactions. The top pane contains O1 (paracetamol) ···H1 (ethanol), the middle pane contains O2 (paracetamol)···H1 (ethanol), and the bottom pane contains H1 (ethanol)···O1 (ethanol) interactions. $N_{2}$ corresponds to the number of molecules of paracetamol in the simulation, which ranges from $x_{2}=0.001$ to $x_{2}=0.115$. The vertical gray line is drawn as a reference and corresponds to the location of the first minimum (i.e., the first solvation shell) for $N_{2}=120$ from Figure 18.

In the Supporting Information accompanying the electronic version of this article, we additionally provide CMat, RDF, and number integral analysis for paracetamol in acetone and DMF (Figures S6– S12) for each concentration studied. The results for acetone and DMF are similar to those for DMSO, with interactions between the solvent ═O (hydrogen bond acceptor) and the hydroxyl (H6) and amide (H1) hydrogens of paracetamol (hydrogen‐bond donor).

## 4 Summary and Conclusion

In this study, we investigated the equilibrium solubility and solution structure of paracetamol in a diverse set of solvents using molecular dynamics simulations. Building upon the work of Belluci et al. [18], we utilized the phase equilibrium relationship Equation (5), that relates the concentration‐dependent solute number density ($\rho_{2}^{L}$) and solvation free energy ($\Delta G_{2}^{\mathrm{solv}}$) in solution, and the extensive excess Gibbs free energy of pure crystalline solute ($G^{S,\mathrm{ex}}$). While $\rho_{2}^{L}$ and $\Delta G_{2}^{\mathrm{solv}}$ are concentration‐dependent properties, we demonstrated that one can efficiently predict the equilibrium solution by staging a series of parallel molecular simulations across a range of concentrations. This approach was leveraged to predict solubility across solvents spanning four orders of magnitude in the experimental concentration.

Our results for ethanol confirmed that the predicted solubility was in excellent agreement with both the experiment and the reference work of Belluci et al. Notably, in highly soluble systems like DMSO, we observed an abrupt change in the linear trend of the solubility expression beyond the predicted equilibrium point. This serves as a thermodynamic signature of a first‐order phase transition, consistent with the expected liquid‐to‐solid transition despite the rarity of structural nucleation events in simulation timescales.

A key methodological finding was the dual utility of the infinite dilution approximation, as shown in Equation (7). While a multi‐concentration regression model is necessary to capture the concentration‐dependent behavior of highly soluble pharmaceuticals such as DMSO and DMF, the infinite dilution approximation is invaluable for initial estimation and for poorly soluble systems. In cases where experimental solubility is unknown, it allows researchers to intelligently stage simulations. Furthermore, it serves as a rapid diagnostic tool; for DEAM, an infinite dilution calculation immediately signals that the model solubility is several orders of magnitude away from experimental values. For solvents with poor equilibrium solubility, such as water and cyclohexane, we found that even at the infinite dilution limit ($N_{2}=1$) within a standard cubic box with edge length of approximately 4.8 nm, the solute chemical potential was already greater than that of the crystalline phase. This indicates that the lowest accessible concentration in such simulations is already supersaturated relative to the model equilibrium, highlighting that extrapolation via the infinite dilution approximation is a necessity rather than merely a convenience. We note that while the simulation concentration is greater than the equilibrium solubility, the system is nonetheless stable as there are no solute–solute interactions in the infinite dilution limit.

The failure of the current force field to model paracetamol in diethylamine (DEAM) highlights a critical distinction: the difference between experimental equilibrium conditions and model equilibrium conditions. While the experimental solubility in DEAM is high (0.3892 mol fracs), the model predicts a value two orders of magnitude lower. As a result, simulations conducted at the experimental concentration, intended to represent equilibrium, such as in the study of nucleation, would actually model a nonphysical metastable state that is not representative of the system of interest. Structural analysis confirmed this discrepancy, identifying that the model underestimates the hydrogen‐bonding strength between the DEAM amine group and the solute, leading to unphysical solute aggregation.

Ultimately, this work demonstrates that applying rigorous phase equilibria expressions is an essential diagnostic in molecular modeling. By utilizing infinite dilution estimates to guide efficient staged experiments and by distinguishing between experimental and model equilibrium, we provide a clear route for the future parameterization of force fields for complex, multifunctional drug‐like molecules.

The failure to capture the high experimental solubility in DEAM provides a clear illustration of the limitations inherent in pure‐component parameterization. This result supports the modern shift toward utilizing condensed–phase mixture data, as seen in recent initiatives like OpenFF Sage 2.0.0 [31], since it demonstrates that even when a force field accurately represents pure component behaviors, it may fail to describe the subtle interspecies associations that dictate solubility in complex amine‐based environments.

Beyond the immediate implications for force field refinement, the identification of specific intermolecular motifs, such as the critical hydrogen‐bonding interactions between paracetamol and DEAM, is vital for the ongoing development of hybrid ML and thermodynamic models. These detailed molecular insights provide the physically grounded descriptors and mechanistic interpretability required to enhance the generalization of data‐driven approaches to novel, complex chemical environments. This work reinforces the role of molecular simulation as an essential source of truth that enables the intelligent design of faster, data‐driven solubility screening tools by pinpointing exactly where classical potentials must be anchored in physical principles to accurately describe multifunctional drug‐like molecules.

## Author Contributions

**Katherine E. Mevis**: formal analysis, investigation, validation, visualization, writing – original draft preparation, writing – review, and editing. **Ella M. Smith**: validation, visualization, writing – original draft preparation, writing – review, and editing. **Andrew S. Paluch**: conceptualization, data curation, formal analysis, funding acquisition, investigation, methodology, project administration, resources, software, supervision, validation, visualization, writing – original draft preparation, writing – review, and editing.

## Conflicts of Interest

The authors declare no conflicts of interest.

## Supporting information

Supplementary Material

Supplementary Material

Supplementary Material

## Data Availability

The data that support the findings of this study are contained in this article.
