# Supplementary material for: Equilibrium Solubility and Solution Structure of Paracetamol: A Molecular Dynamics Study Spanning Four Orders of Magnitude in Diverse Pure Solvents
Source: Chemphyschem. 2026 Oct 22;27(20):e70593. doi: 10.1002/cphc.70593 (PMC13691287; doi:10.1002/cphc.70593)
Supplement: Supplementary file 1 [file CPHC-27-e70593-s001.pdf]

Supporting Information for: Equilibrium Solubility and Solution Structure of Paracetamol: A Molecular Dynamics Study Spanning Four Orders of Magnitude in Diverse Pure Solvents

Katherine E. Mevis[a], Ella M. Smith[a], Andrew S. Paluch[a]

[a]*Department of Chemical, Paper and Biomedical Engineering, Miami University, Oxford, Ohio 45056, USA*

Email address: PaluchAS@MiamiOH.edu (Andrew S. Paluch)

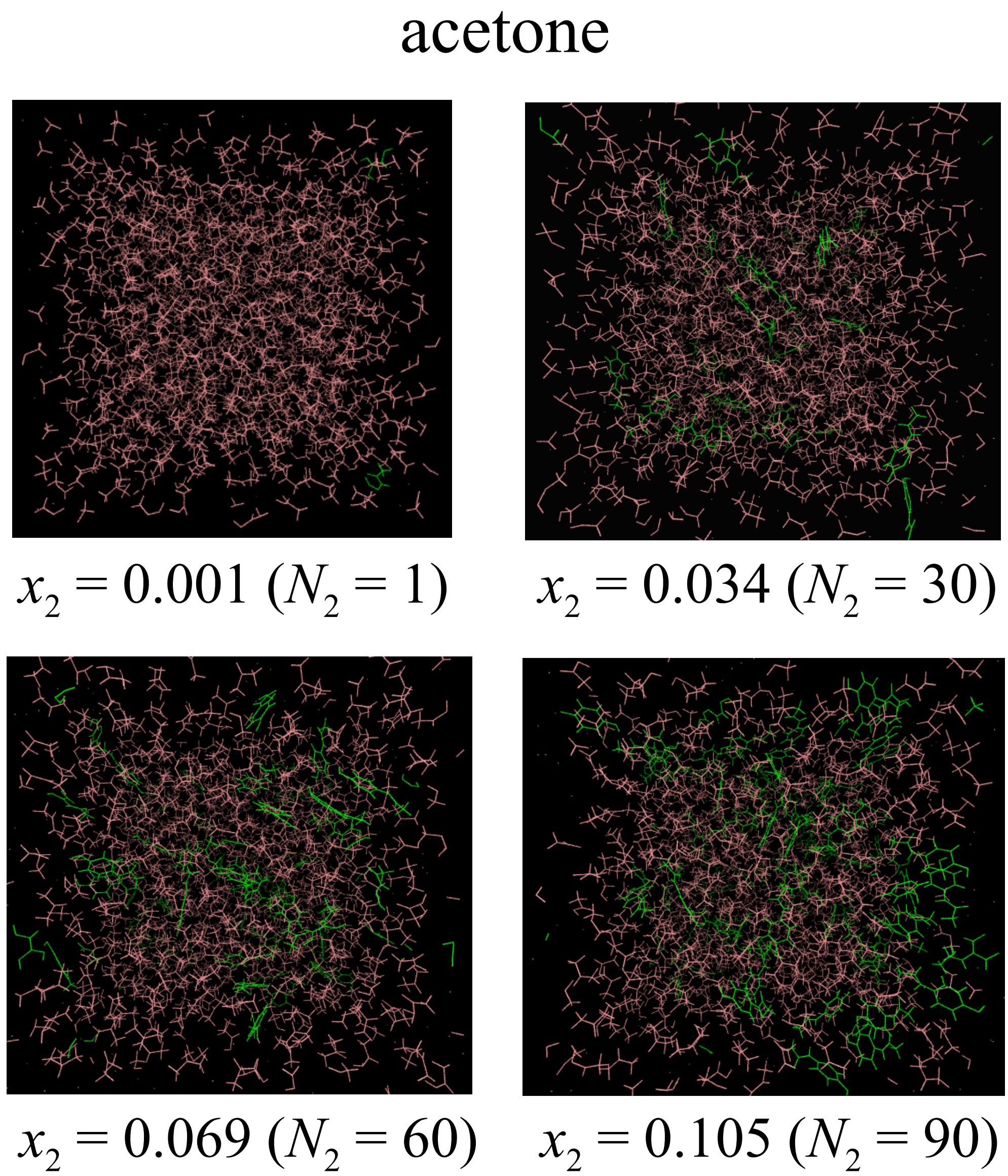

Figure S1: A snapshot of the central simulation cell for paracetamol in acetone at each concentration studied, as indicated. Green and rust are used to represent paracetamol and acetone molecules, respectively, and x_2 and N_2 correspond to the mole frac and number of paracetamol molecules, respectively.

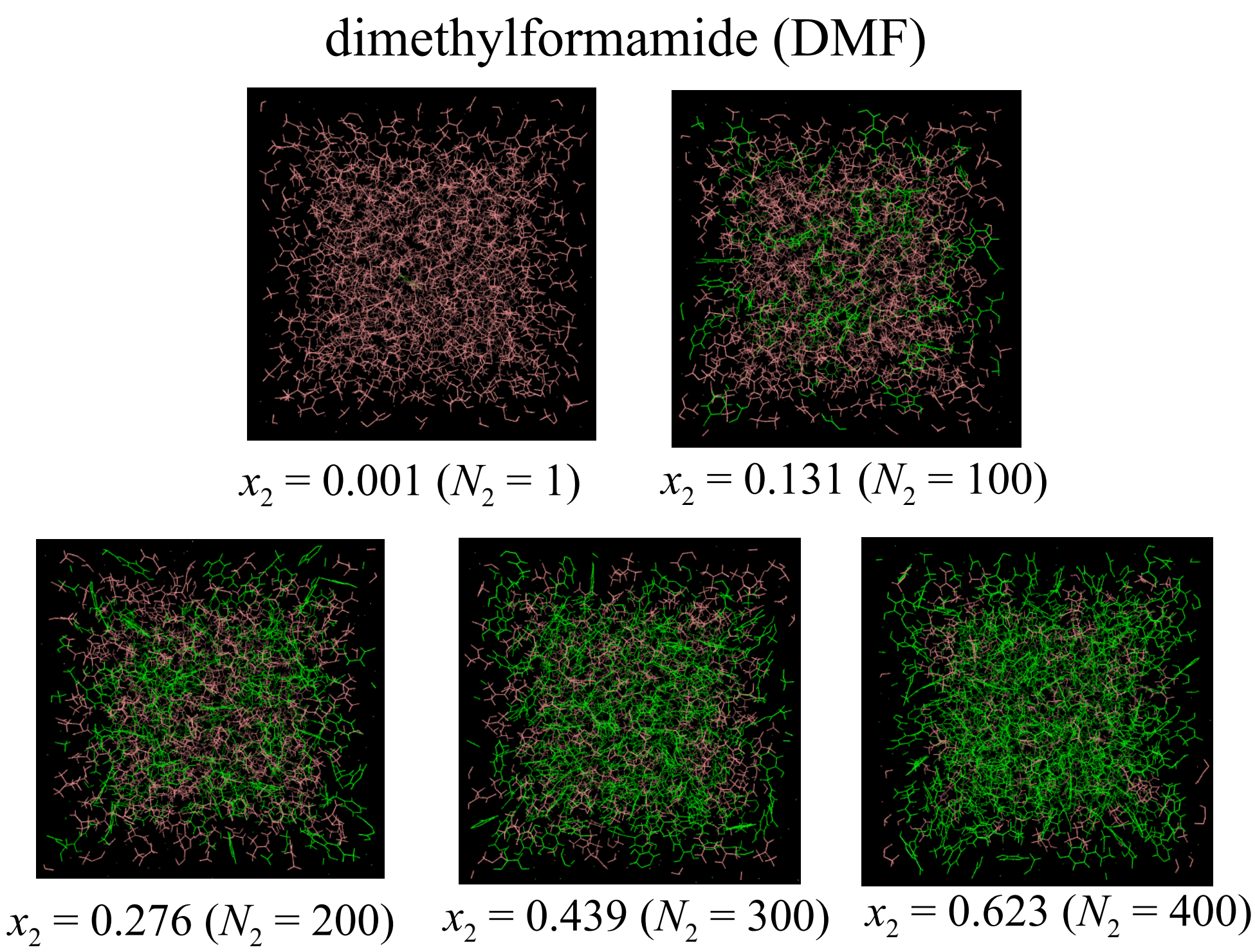

Figure S2: A snapshot of the central simulation cell for paracetamol in dimethylformamide (DMF) at each concentration studied, as indicated. Green and rust are used to represent paracetamol and DMF molecules, respectively, and x_2 and N_2 correspond to the mole frac and number of paracetamol molecules, respectively.

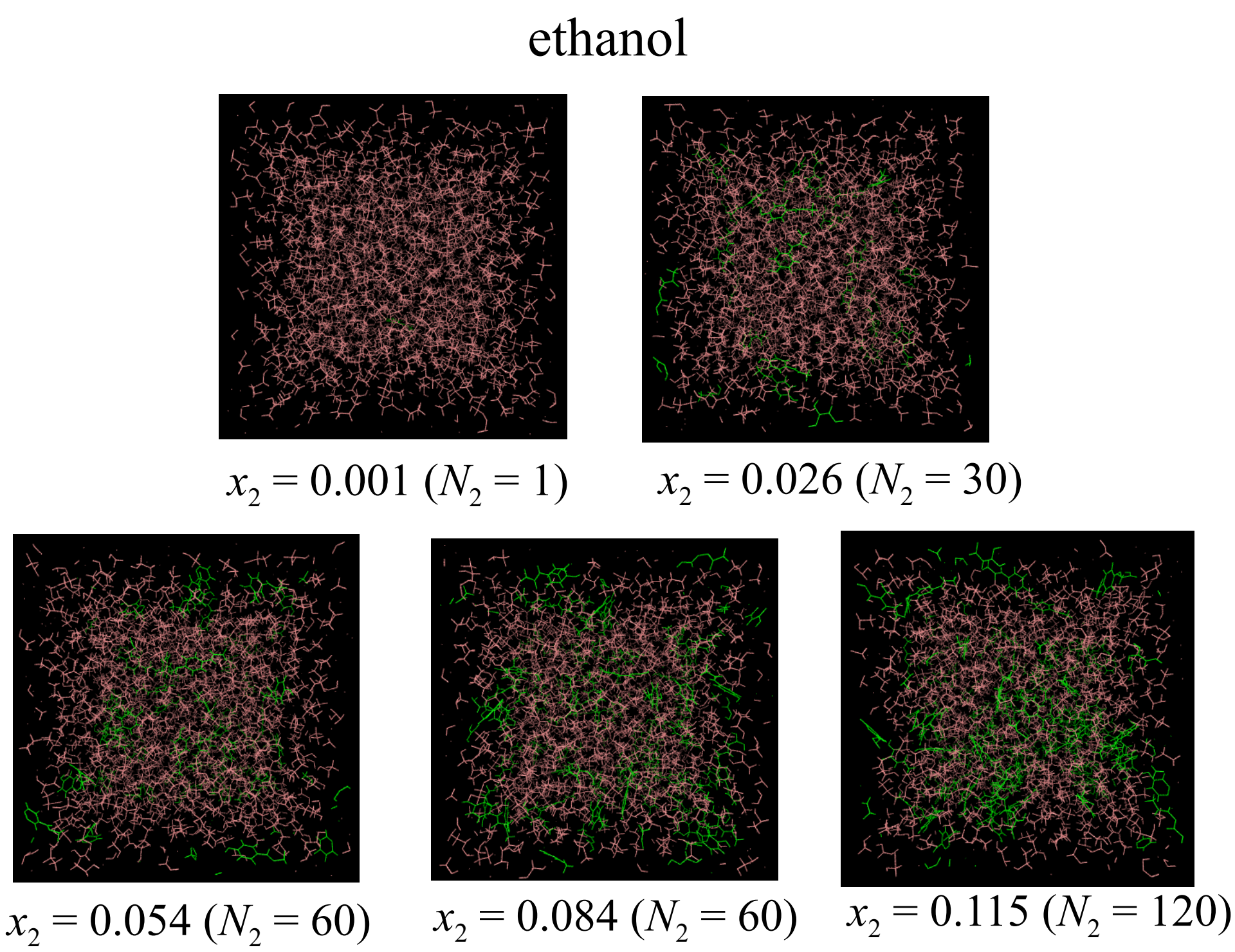

Figure S3: A snapshot of the central simulation cell for paracetamol in ethanol at each concentration studied, as indicated. Green and rust are used to represent paracetamol and ethanol molecules, respectively, and x_2 and N_2 correspond to the mole frac and number of paracetamol molecules, respectively.

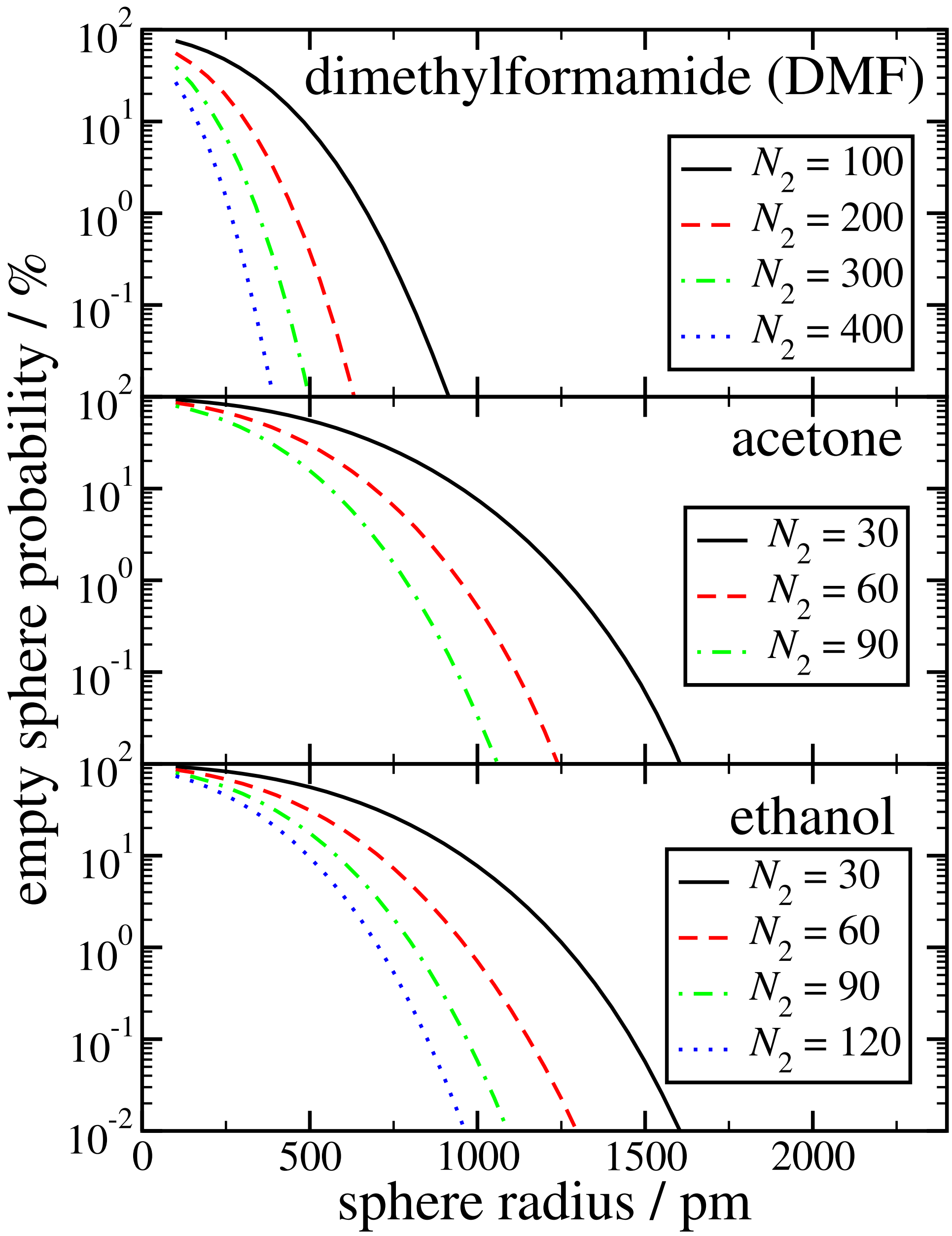

Figure S4: Plot of the probability of finding an empty sampling sphere as a function of sphere radius for paracetamol in DMF (top pane), acetone (middle pane), and ethanol (bottom pane) at each concentration studied ($N_2 > 1$, see figures S2, S1 and S3).

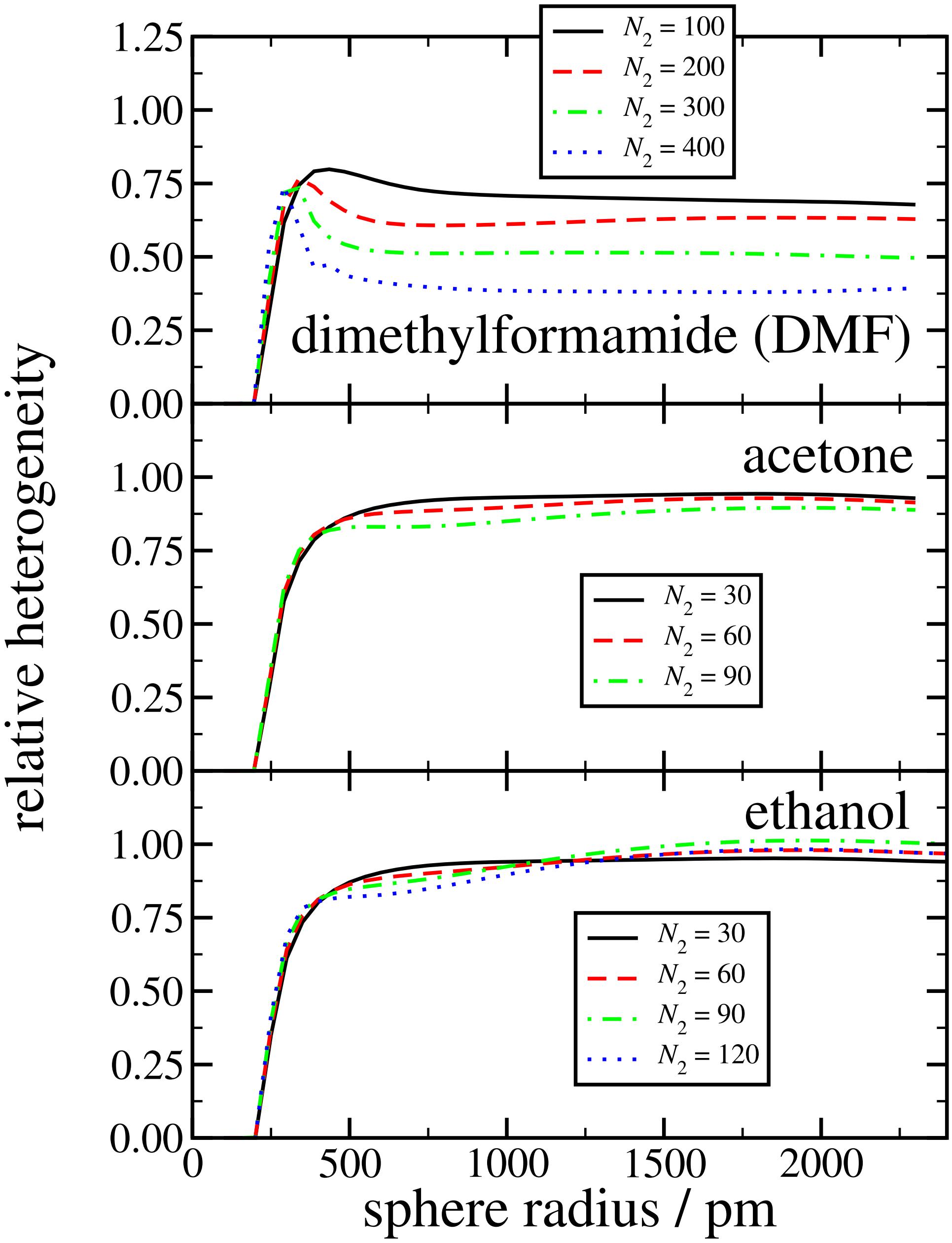

Figure S5: Plot of the relative heterogeneity index (h_{rel}) as a function of sphere radius for paracetamol in DMF (top pane), acetone (middle pane), and ethanol (bottom pane) at each concentration studied ($N_2 > 1$, see figures S2, S1 and S3).

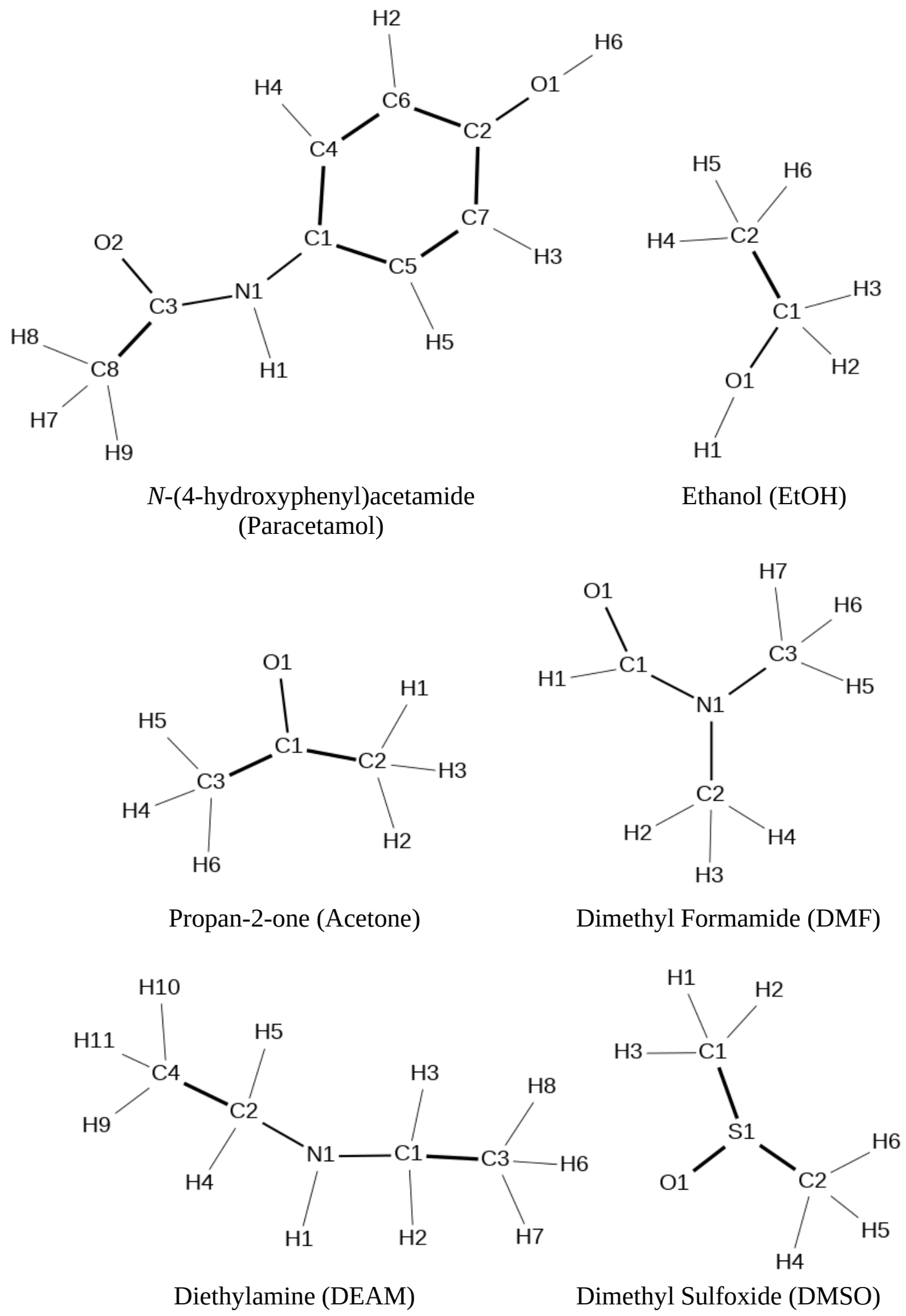

Figure S6: Chemical structure of paracetamol, ethanol (EtOH), propan-2-one (acetone), dimethylformamide (DMF), diethylamine (DEAM), and dimethyl sulfoxide (DMSO) and the labeling scheme used when performing structural analysis.

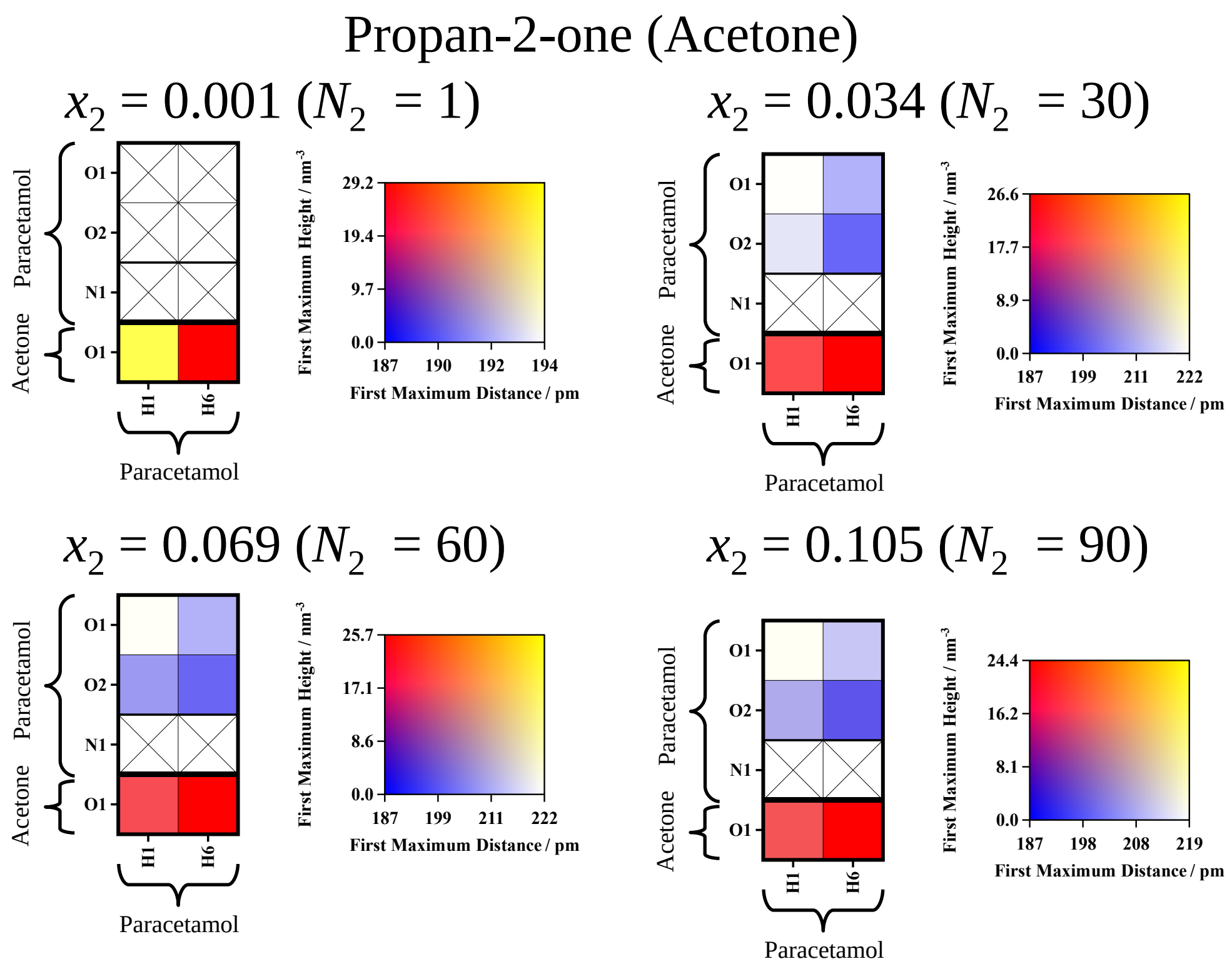

Figure S7: Connection matrix analysis for paracetamol in propan-2-one (acetone) for each concentration studied. The rows represent hydrogen bond acceptors, and the columns represent hydrogen bond donors. The colors represent the intensity and distance of the first maximum in the local density, $\rho_{i,j}\left(r_{i,j}\right)$ (i.e., the un-normalized radial distribution function), as indicated in the scale on the right, and x_2 and N_2 correspond to the mole frac and number of paracetamol molecules, respectively. The presence of a black cross indicates no interaction was detected within a distance of 350 pm.

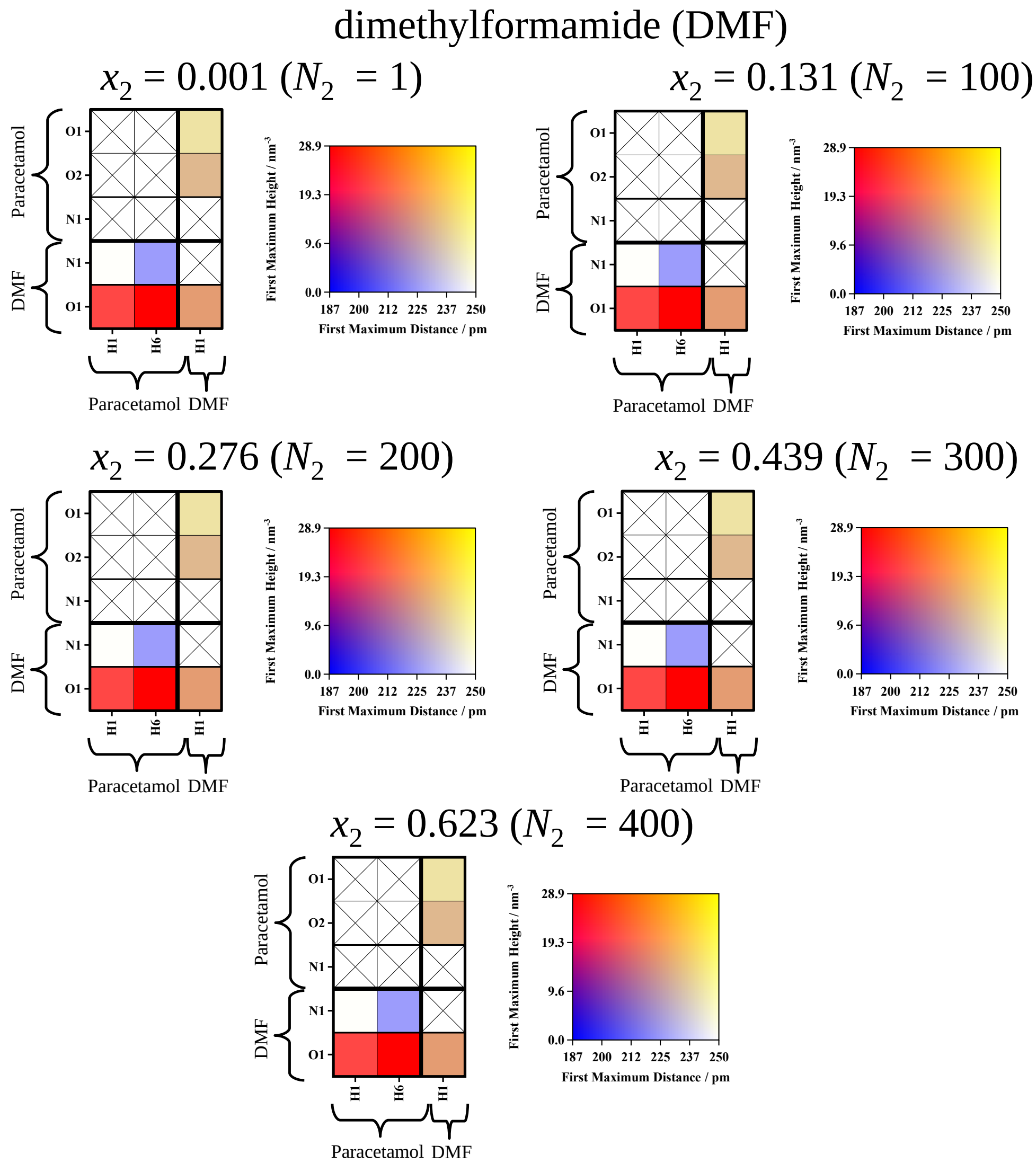

Figure S8: Connection matrix analysis for paracetamol in dimethylformamide (DMF) for each concentration studied. The rows represent hydrogen bond acceptors, and the columns represent hydrogen bond donors. The colors represent the intensity and distance of the first maximum in the local density, $\rho_{i,j}\,(r_{i,j})$ (i.e., the un-normalized radial distribution function), as indicated in the scale on the right, and x_2 and N_2 correspond to the mole frac and number of paracetamol molecules, respectively. The presence of a black cross indicates no interaction was detected within a distance of 350 pm.

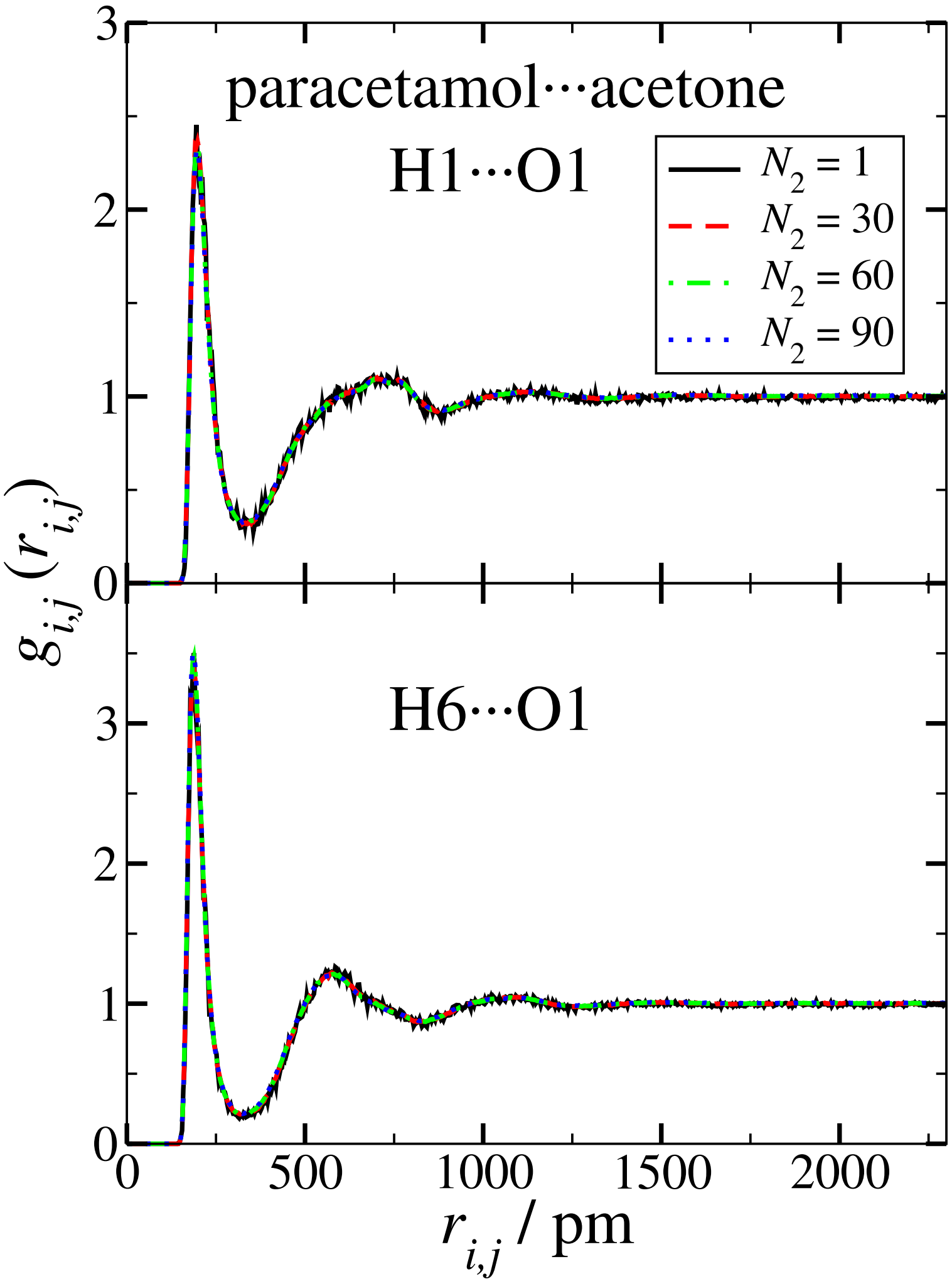

Figure S9: Radial distribution function analysis, $g_{i,j}$, for paracetamol$\cdots$propan-2-one (acetone) interactions. The top pane contains H1 (paracetamol)$\cdots$O1 (acetone) and the bottom pane contains H6 (paracetamol)$\cdots$O1 (acetone) interactions. N_2 corresponds to the number of molecules of paracetamol in the simulation, which ranges from $x_2 = 0.001$ to $x_2 = 0.105$. For H1$\cdots$O1 the intensity of the first maximum changes little going from $N_2 = 1$ to $N_2 = 90$ (2.454 to 2.326), while for H6$\cdots$O1 there is a noticeable increase (1.195 to 3.506).

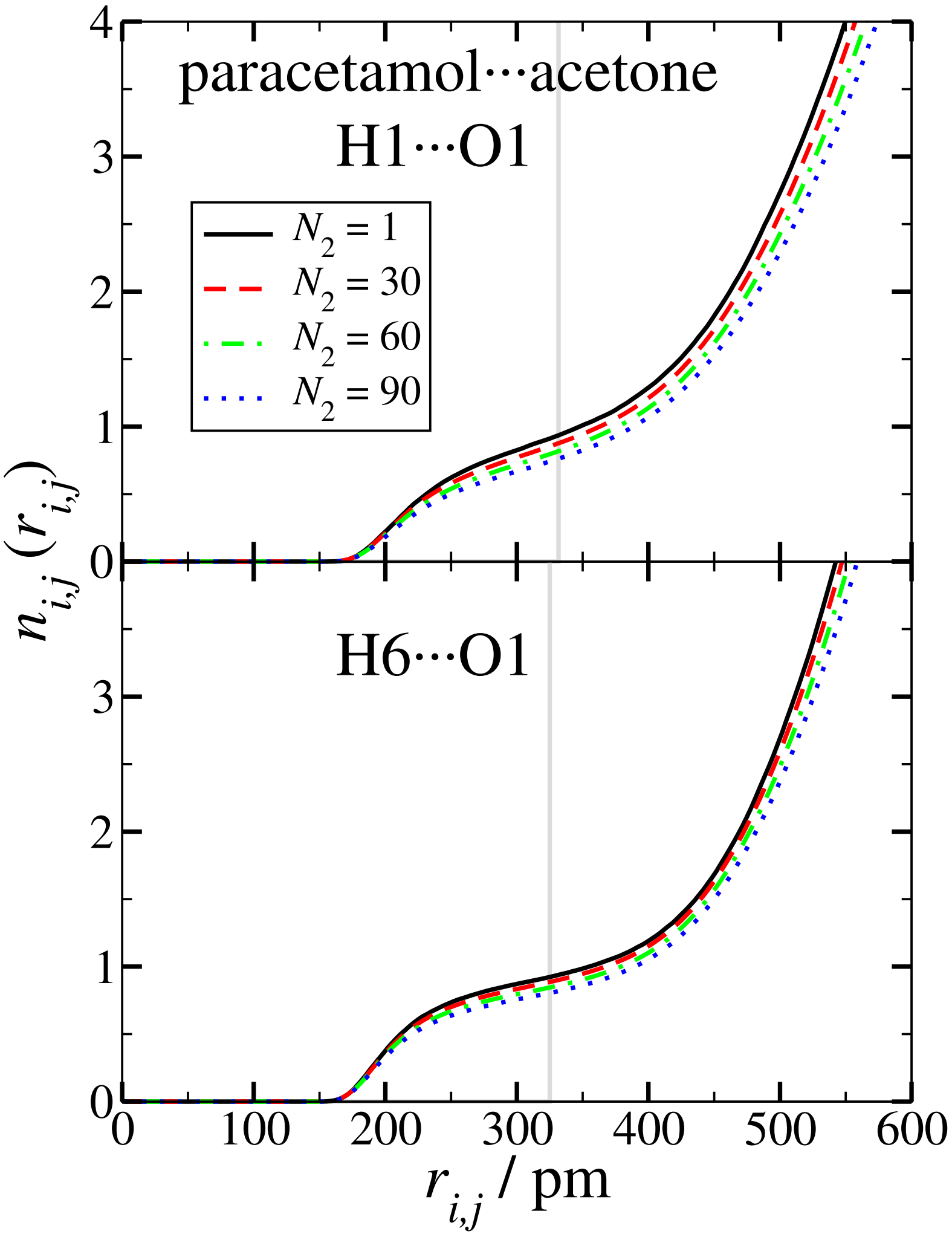

Figure S10: Number integral analysis, $n_{i,j}$, for paracetamol$\cdots$propan-2-one (acetone) interactions, complementary to figure S9. The top pane contains H1 (paracetamol)$\cdots$O1 (acetone) and the bottom pane contains H6 (paracetamol)$\cdots$O1 (acetone) interactions. N_2 corresponds to the number of molecules of paracetamol in the simulation, which ranges from $x_2 = 0.001$ to $x_2 = 0.105$. The vertical grey line is drawn as a reference and corresponds to the location of the first minimum (i.e., the first solvation shell) for $N_2 = 90$ from figure S9.

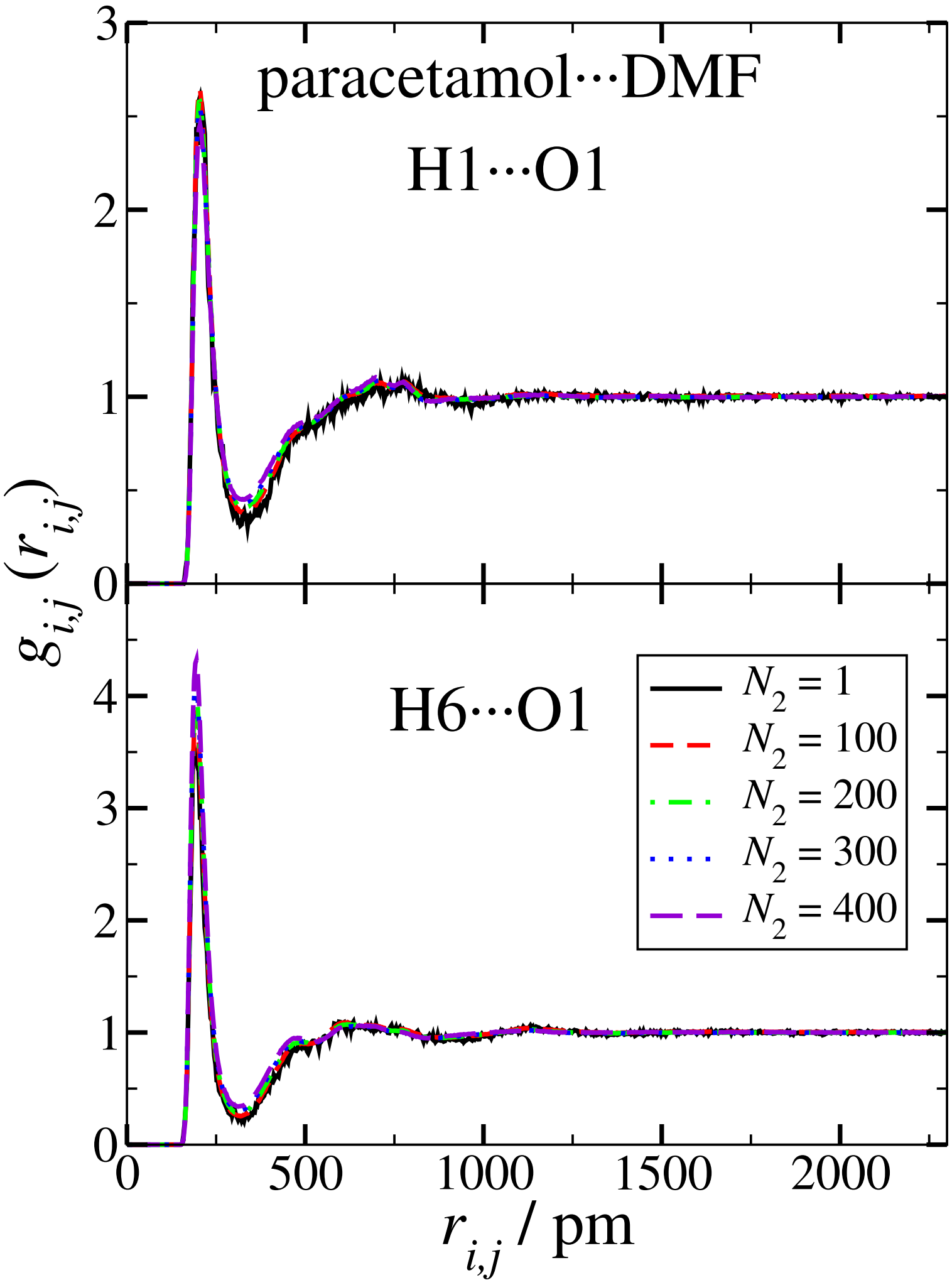

Figure S11: Radial distribution function analysis, $g_{i,j}$, for paracetamol$\cdots$dimethylformamide (DMF) interactions. The top pane contains H1 (paracetamol)$\cdots$O1 (DMF) and the bottom pane contains H6 (paracetamol)$\cdots$O1 (DMF) interactions. N_2 corresponds to the number of molecules of paracetamol in the simulation, which ranges from $x_2 = 0.001$ to $x_2 = 0.623$. For H1$\cdots$O1 the intensity of the first maximum changes little going from $N_2 = 1$ to $N_2 = 400$ (2.603 to 2.455), while for H6$\cdots$O1 there is a slight increase (3.535 to 4.322).

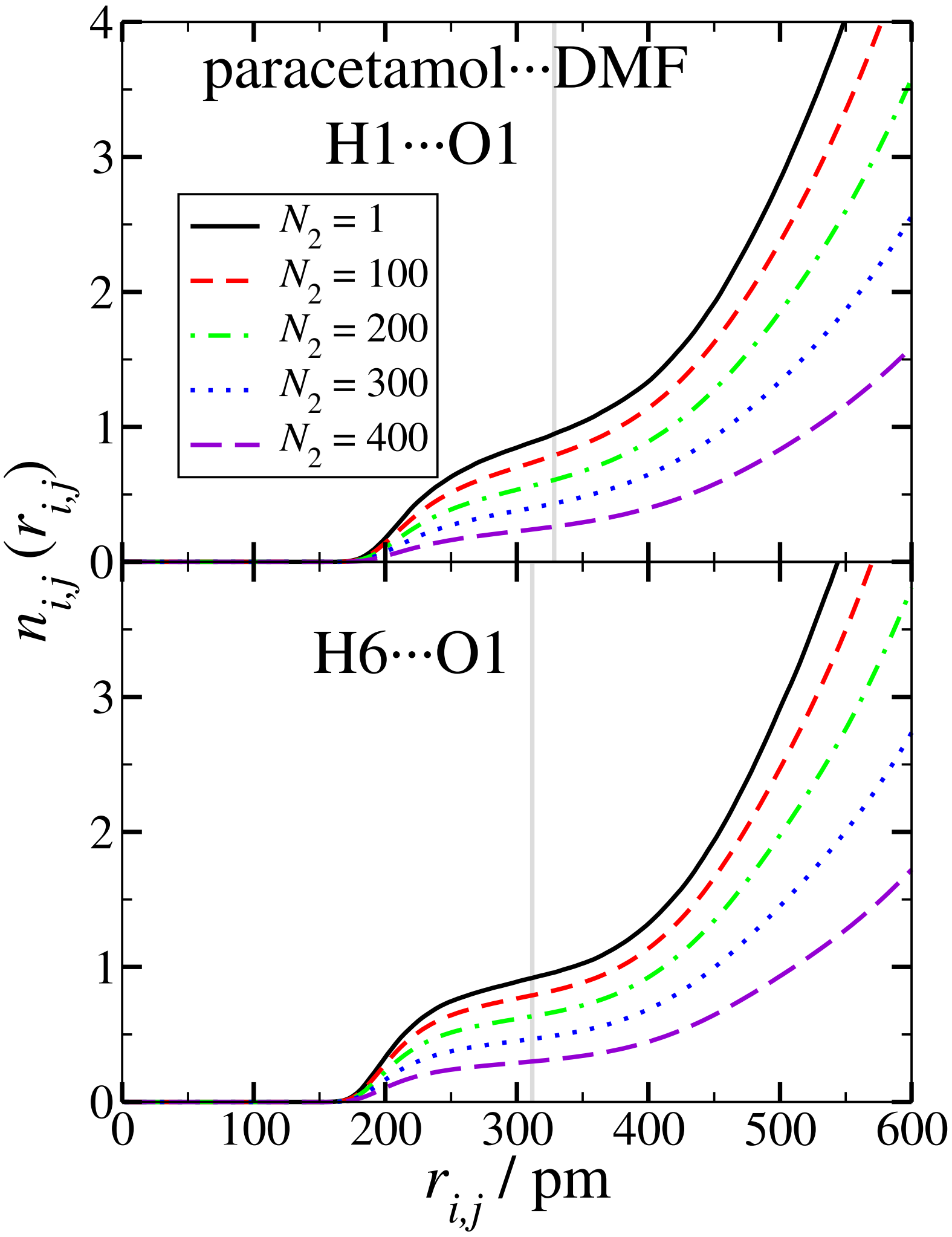

Figure S12: Number integral analysis, $n_{i,j}$, for paracetamol$\cdots$dimethylformamide (DMF) interactions, complementary to figure S11. The top pane contains H1 (paracetamol)$\cdots$O1 (DMF) and the bottom pane contains H6 (paracetamol)$\cdots$O1 (DMF) interactions. N_2 corresponds to the number of molecules of paracetamol in the simulation, which ranges from $x_2 = 0.001$ to $x_2 = 0.623$. The vertical grey line is drawn as a reference and corresponds to the location of the first minimum (i.e., the first solvation shell) for $N_2 = 400$ from figure S11.
